# Structural Bases of Dihydroxy Acid Dehydratase Inhibition and Biodesign for Self-Resistance

**DOI:** 10.34133/bdr.0046

**Published:** 2024-11-01

**Authors:** Xin Zang, Undramaa Bat-Erdene, Weixue Huang, Zhongshou Wu, Steve E. Jacobsen, Yi Tang, Jiahai Zhou

**Affiliations:** ^1^ Shenzhen Institute of Synthetic Biology, Shenzhen Institute of Advanced Technology, Chinese Academy of Sciences, Shenzhen 518055, China.; ^2^ Department of Chemical and Biomolecular Engineering, University of California, Los Angeles, CA, USA.; ^3^ State Key Laboratory of Chemical Biology, Shanghai Institute of Organic Chemistry, Chinese Academy of Sciences, Shanghai 200032, China.; ^4^ Department of Molecular Cell and Developmental Biology, University of California, Los Angeles, CA 90095, USA.; ^5^ Howard Hughes Medical Institute, University of California, Los Angeles, CA 90095, USA.; ^6^ Eli and Edythe Broad Center of Regenerative Medicine and Stem Cell Research, University of California, Los Angeles, CA 90095, USA.; ^7^ Department of Biological Chemistry, University of California, Los Angeles, CA 90095, USA.; ^8^ School of Food Science and Pharmaceutical Engineering, Nanjing Normal University, Nanjing 210023, China.

## Abstract

Dihydroxy acid dehydratase (DHAD) is the third enzyme in the plant branched-chain amino acid biosynthetic pathway and the target for commercial herbicide development. We have previously reported the discovery of fungal natural product aspterric acid (AA) as a submicromolar inhibitor of DHAD through self-resistance gene directed genome mining. Here, we reveal the mechanism of AA inhibition on DHAD and the self-resistance mechanism of AstD, which is encoded by the self-resistance gene *ast*D. As a competitive inhibitor, the hydroxycarboxylic acid group of AA mimics the binding of the natural substrate of DHAD, while the hydrophobic moiety of AA occupies the substrate entrance cavity. Compared to DHAD, AstD has a relatively narrow substrate channel to prevent AA from binding. Several mutants of DHAD were generated and assayed to validate the self-resistance mechanism and to confer *Arabidopsis thaliana* DHAD with AA resistance. These results will lead to the engineering of new type of herbicides targeting DHAD and provide direction for the ecological construction of herbicide-resistant crops.

## Introduction

**Fig. 1.**
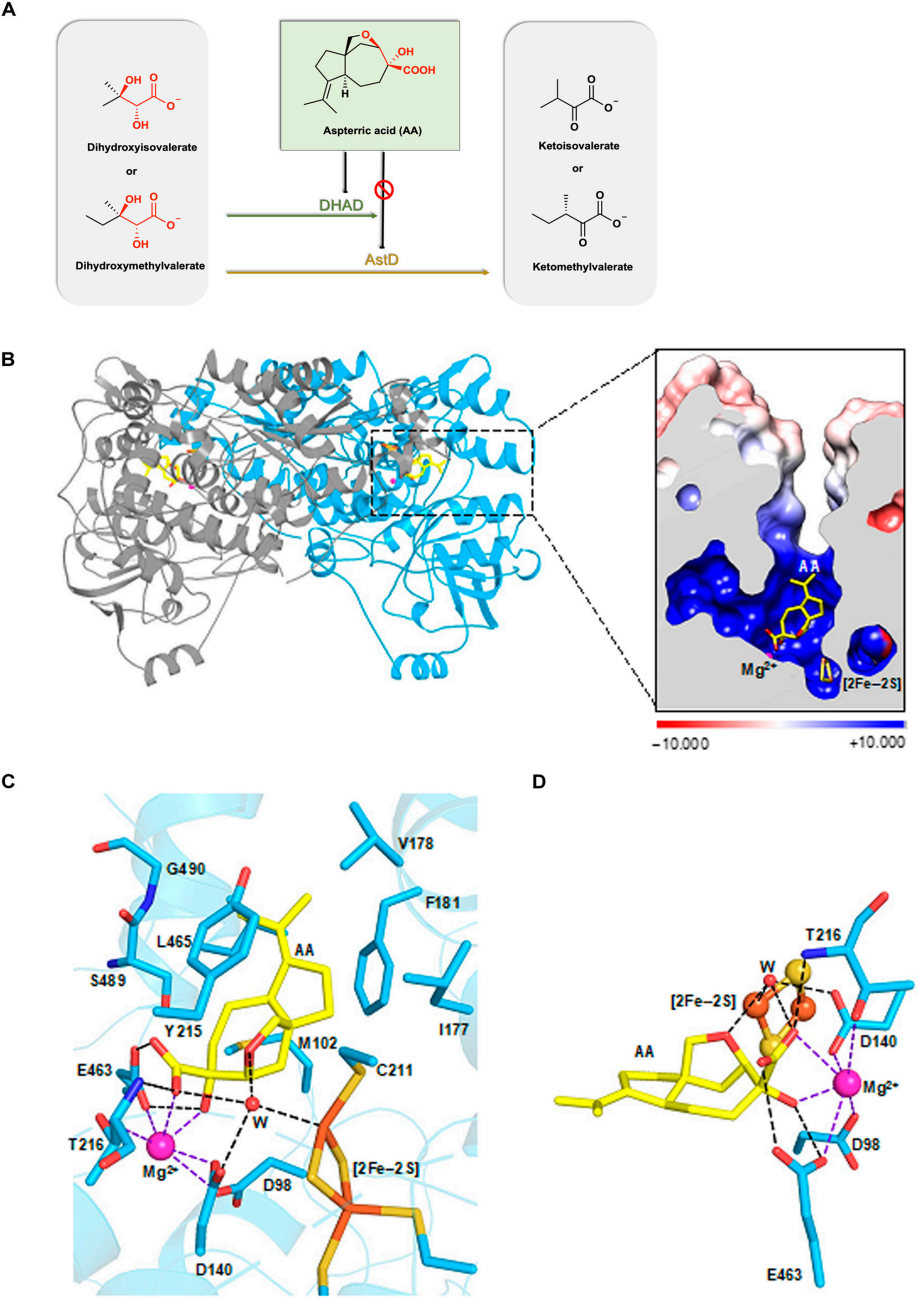
Crystal structure of Holo-DHAD complexed with AA. (A) The reaction catalyzed by DHAD in *A.thaliana* and AstD (SRE in *Aspergillus terreus*) in BCAA. AA can inhibit *Ath*DHAD but cannot inhibit AstD. (B) Dimer structure of *Ath*DHAD (the 2 chains are in blue and gray, respectively) and the AA binding pocket. AA is shown in yellow. The surface was generated by using the APBS (Adaptive Poisson-Boltzmann Solver) algorithm at pH 7.0. The positive and negative charges are colored blue and red, respectively, according to the scale [27,28]. (C) The binding mode of AA to *Ath*DHAD. Hydrogen bonds and coordination bonds are shown as black dash lines and purple dash lines, respectively. Water is shown as sphere in red. (D) The polar interactions between AA and *Ath*DHAD, including the coordination and hydrogen bonds.

The use of herbicides has greatly increased crop yields. For example, from 1964 to 1979, yields of corn and soybean in the United States were increased by 20% and 62%, respectively, owing to the use of commercial herbicides [1,2]. Simultaneously, herbicide-resistant weeds started emerging since the introduction of herbicides in mid-1940s [3]. Over the last 4 decades, few herbicides with new modes of action have been introduced to the market, and the repeated use of herbicides exacerbated the problem of herbicide-resistant weeds [4–6]. The continuous rise of herbicide resistance is posing threat to the food supply for humans [7–10]. For example, while acetolactate synthase, the first common enzyme in the branched-chain amino acid (BCAA) biosynthetic pathway, is the most targeted enzyme for commercial herbicide development with about 56 products, all are ineffective toward weeds containing a widely found resistance mutation [11–14]. Herbicides with new modes of action are urgently needed for crop production. Toward this goal, we previously described the fungal sesquiterpene natural product aspterric acid (AA) as a submicromolar inhibitor of plant dihydroxy acid dehydratase (DHAD), the third common enzyme in the BCAA pathway (Fig. 1A) [8]. Our discovery of AA emerged from a resistance gene-guided genome mining effort that leveraged the following: (a) strong sequence identity between fungal and plant housekeeping DHAD; (b) the requirement for fungal producers of an inhibitor to also encode a self-resistance enzyme (SRE) that is similar in sequence to the housekeeping DHAD but is insensitive to said inhibitor; and (c) genes encoding the SRE and the biosynthetic enzymes are colocalized as a biosynthetic gene cluster and can be bioinformatically identified.

In our previous work, using the sequence of DHAD as a probe, we discovered a highly conserved set of 4 genes across various fungal genomes, including the well-known soil fungus *Aspergillus terreus*, recognized for its production of lovastatin. These conserved gene clusters consist of a gene encoding a sesquiterpene cyclase homolog (astA), 2 cytochrome P450 genes (astB and astC), and a homolog of DHAD (astD), which is potentially an SRE.

The enzymes encoded by *ast*ABC were responsible for the synthesis of AA. AA was confirmed to be a competitive inhibitor of housekeeping DHAD from both *A. terreus* (XP_001208445.1, *Ate*DHAD) and *A. thaliana* (AT3G23940, *Ath*DHAD) but not of AstD. AA was effective as an herbicide in spray experiments, using *A. thaliana* as a model plant organism. Transgenic *A. thaliana* expressing AstD showed strong resistance to AA, thereby establishing a compound/resistance gene pair that may be attractive for further herbicide development [15]. Although we reported the structure of the *Ath*DHAD in the same work (Protein Data Bank [PDB] ID: 5ZE4), the lack of DHAD–AA complex structure prevents the elucidation of the mechanism by which AA inhibits DHAD, hindering further herbicide designs for this novel target.

In this work, we solved the crystal structure of *Ath*DHAD–AA complex with 2.0-Å resolution, which provides the critical information for the inhibition mechanism at the atomic level. Here, we report the full interaction of AA with *Ath*DHAD, including hydroxycarboxylic acid with Mg^2+^, the hydrogen bonding networks, and the extensive hydrophobic interactions in the active site of *Ath*DHAD. We also determined the crystal structure of AstD at 2.3-Å resolution. The 2Fe–2S cluster was docked since it was lost during the crystallization. The AstD substrate entrance channel is narrower than that of *Ath*DHAD, explaining its resistance to AA. Guided by the structural difference between AstD and *Ath*DHAD, several mutants of *Ath*DHAD were constructed. They acquired moderate tolerance to AA while retaining the original activity. These results will contribute to the development of new herbicides targeting DHAD and the establishment of new weed management system.

## Materials and Methods

### Strains and general culture conditions

To facilitate the production of AA and enable genomic DNA or mRNA extractions, *Aspergillus terreus* NIH 2624 was grown for 14 d at a constant 28°C in stationary liquid potato dextrose broth medium (Fisher Scientific). We employed the *Escherichia coli* BL21(DE3) strain from Novagen for heterologous protein expression. Our *E. coli* strains were cultivated in lysogeny broth (LB)  media, maintained at 37°C for cloning purposes and at a cooler 16°C for protein expression.

### General DNA manipulation techniques

Following strict adherence to manufacturers’ protocols, we conducted all DNA manipulations. We utilized DNA restriction enzymes from New England Biolabs as per the supplier’s recommendations, while polymerase chain reaction (PCR) amplifications were performed using New England Biolabs’s Q5 High-Fidelity DNA polymerase, following their specified procedures. The primers employed are listed in Table S1. Verification of PCR products and plasmid DNAs was achieved through DNA sequencing and restriction enzyme digestion checks. For cloning purposes, we used the *E. coli* TOP10 strain.

### Generating AstD and mutant *Ath*DHAD plasmids for in vitro assays and crystallization

To generate an intron-free open reading frame of the *AthDHAD* gene, we began by using a gBlock template for amplification via PCR, employing primers (detailed in Table S1) specifically designed to append a 6-histidine tag to the C-terminus. The resulting PCR product was seamlessly integrated into a linearized pET28a vector through a process of digestion and ligation, adhering to the manufacturer’s protocol. This process yielded the plasmid pEUB1000. To introduce specific amino acid mutations, we used pEUB10001 as the template. Using primers listed in Table S1, we performed PCR amplification of the entire plasmid. Following this, the plasmids underwent sequencing to confirm the presence of the desired mutations, ensuring accuracy and correctness in our genetic modifications.

Plasmids used for expressing *Ath*DHAD and its mutations for crystallization were constructed using the methods described in a previously published article [15]. The double mutant K559A/K560A for efficient crystallization was designed using the surface entropy reduction prediction server [16]. The encoding sequence of AstD (residues 43 to 598) was cloned into pSJ2 with an 8-histidine (8xHis) tag and a tobacco etch virus protease cleavage site at the N-terminus.

### Large-scale AA isolation

For the large-scale isolation of AA, *Aspergillus terreus* was cultivated in 6 l of stationary liquid potato dextrose broth media for 14 d. Following this period, the culture was filtered through cloth to separate the cell mass from the culture liquid. The cell mass underwent 3 times of extractions with 1 l of acetone each, and the resulting extracts were concentrated using a Buchi Rotavapor under reduced pressure. Meanwhile, the organic solutes present in the culture liquid were extracted 3 times with an equal volume of ethyl acetate, which were then dried under reduced pressure. Both sets of extracts—from the cell mass and the culture liquid—were combined, yielding a crude extract oil. This crude extract was first purified using a normal-phase silica column on a CombiFlash system with hexane. The compound underwent further refinement using a reversed-phase CombiFlash system. This involved a linear gradient of 5% to 95% methanol (MeOH)/water with 0.1% formic acid over a period of 35 min, followed by 95% MeOH for 10 min, all at a flow rate of 6 ml/min through a 40-g silica column. The fractions containing AA were combined and then further purified via high-performance liquid chromatography (HPLC). This purification utilized a semipreparative reversed-phase column with a gradient of 65% to 100% acetonitrile/water over 25 min at a flow rate of 2.5 ml/min. The final purification step was conducted using a Shimadzu Prominence HPLC system, equipped with a Phenomenex Kinetex, 5μ, 10.0 × 250 mm, C-18 column.

### Protein expression and purification for in vitro assays and crystallization

Enzymes utilized in vitro assays underwent a meticulous process of expression and purification. Initially, plasmids encoding *Ath*DHAD and its variants were individually introduced into *E. coli* BL21(DE3) and cultured overnight in 5 ml of LB medium supplemented with 50 μg/ml kanamycin at 37°C. These overnight cultures served as inoculants for 1 l of fresh LB media, also containing 50 μg/ml kanamycin, incubated until reaching an optical density at 600 nm (OD_600_) of 0.6 at 37°C. Upon reaching this point, the cultures were chilled on ice, and protein overexpression was initiated by adding 0.1 mM isopropyl-β-D-thiogalactopyranoside (IPTG, GoldBio, USA), followed by overnight incubation at 16°C. Simultaneously, 125 μM FeSO_4_ was introduced into the cultures with IPTG induction. Subsequently, *E. coli* cells were harvested by centrifugation at 5,300 rpm for 15 min and resuspended in 30 ml of A10 buffer (50 mM tris-HCl and 10 mM imidazole [pH 8.0]) supplemented with 1 tablet of Pierce protease inhibitor (Thermo Scientific). The cell suspension underwent sonication on ice, followed by centrifugation at 17,000 g for 15 min at 4°C to eliminate insoluble cellular debris. The resulting soluble fractions containing recombinant C-terminally hexahistidine-tagged *Ath*DHADs were subjected to individual purification using affinity chromatography with Ni-nitrilotriacetic acid agarose resin. The Ni-sepharose suspension and lysate were incubated at 4°C for 2 h, followed by sequential washing with 50 ml of A10 buffer and 25 ml of A25 buffer. Elution of proteins was achieved using 1.5 ml of A250 buffer, devoid of NaCl, and the purified enzymes were directly employed for assays. Verification of protein purity was conducted through sodium dodecyl sulfate-polyacrylamide gel electrophoresis analysis, while protein concentration was determined via Bradford Protein Assay (Bio-Rad), utilizing bovine serum albumin (Sigma-Aldrich) as a standard.

Proteins used for crystallization were expressed and purified as below. The plasmids PDB1281 was cotransformed with plasmids encoding *Ath*DHAD for Fe–S cluster production. *E. coli* BL21(DE3) was used for protein expression. The cells were inoculated to 5 ml of LB medium with kanamycin (30 μg/ml) and ampicillin (100 μg/ml) growing 6 h at 37°C while being shaken constantly at 150 rpm. Then the culture was transferred to 1 l of LB medium with 30 μg/ml kanamycin and 100 μg/ml ampicillin. The cells were growing at 37°C for 4 h and were induced with 0.1 mM IPTG, 0.1% L-arabinose, and 1 mM FeCl_3_ at an OD_600_ of 1.0 when it cooled to 16°C. After incubating for 16 h, the culture was harvested at 8,000 rpm for 3 min at 4°C. AstD was expressed by the same method. The purification procedures were operated under aerobic conditions. The cell pellet was resuspended in buffer A (25 mM tris [pH 8.0], 500 mM NaCl, and 1 mM TCEP) and lysed by sonication on ice (Shanghai Union Biotech). Cell debris was removed by centrifugation at 18,000 rpm, 4°C for 20 min. Recombinant proteins in the supernatant were purified using nickel-sepharose resin (GE Healthcare) and eluted stepwise with imidazole. TEVTobacco etch virus protease was used to remove the fusion his-tag in dialysate buffer (50 mM tris [pH 8.0], 50 mM NaCl, and 1 mM TCEP) about 2 h. The protein mixture was then reloaded onto a 5-ml Ni-nitrilotriacetic acid column, and flow-through was collected. The purified proteins were concentrated in a solution with 25 mM tris (pH 8.0), 150 mM NaCl, and 2 mM DTT.

### Protein crystallization

The proteins were subjected to crystallization within an anaerobic environment. Initially, proteins at a concentration of 10 mg/ml were combined in a 1:1 ratio with the reservoir solution, with a total volume of 1 μl. This mixture was then equilibrated against a 50-μl reservoir solution utilizing the sitting-drop vapor diffusion method and maintained at 16°C. *Ath*DHAD was incubated with AA in a ratio of 1:5 before crystallization. The crystals of the *Ath*DHAD–AA complex, *Ath*DHAD–I177F, *Ath*DHAD–V178W, *Ath*DHAD–V496W, and *Ath*DHAD–V497F were observed in the condition of 0.1 M sodium acetate (pH 5.0) and 1.5 M ammonium sulfate after 3 d. The best crystals of AstD were optimized under condition of 1× MMT buffer (200 mM DL-malic acid, 430 mM MES, and 400 mM tris [pH 4.0]), 17% polyethylene glycol 1500, and 1 mM TCEP (pH 4.0) after 7 d.

### Protein crystal data collection and processing

All crystals were flash-cooled in liquid nitrogen after cryo-protected with crystallization solution containing 25% glycerol. The *Ath*DHAD–AA complex data were collected at 100 K using Beam Line 19U1 at the Shanghai Synchrotron Radiation Facility (SSRF), with diffraction data recorded at a wavelength of 0.97852 Å. The highest-quality crystals provided a resolution of 2.0 Å. According to the Ramachandran plot, 98.06% of residues were in favored regions, 1.76% in allowed regions, and 0.18% in outlier regions. Data processing, including indexing, integration, and scaling, was carried out using the XDS package [17]. These crystals were classified under space group P42212, with detailed data collection statistics provided in Table 1.

**Table 1.** X-ray data collection and refinement statistics

	*Ath*DHAD–AA (PDB 9JPI)	AstD (PDB 9IX7)	*Ath*DHAD–V496W (PDB 8IKZ)	*Ath*DHAD–V497F (PDB 8IMU)	*Ath*DHAD–I177F (PDB 9JSQ)	*Ath*DHAD–V178W (PDB 8HS0)
**Data collection**						
Space group	*P4_2_2_1_2*	*P2_1_2_1_2_1_*	*P4_2_2_1_2*	*P4_1_2_1_2*	*P4_2_2_1_2*	*P4_2_2_1_2*
Cell dimensions						
*a*, *b*, *c* (Å)	135.8, 135.8, 66.64	56.61, 66.27, 268.9	135.6, 135.6, 66.74	135.1, 135.1, 136.4	135.5, 135.5, 66.18	135.9 135.9 66.38
α, β, γ (°)	90.00, 90.00, 90.00	90.00, 90.00, 90.00	90.00, 90.00, 90.00	90.00, 90.00, 90.00	90.00, 90.00, 90.00	90.00, 90.00, 90.00
Resolution (Å)	47.58–2.00 (2.07–2.00) ^a^	50–2.30 (2.34–2.30) ^a^	47.53–1.75 (1.78–1.75) ^a^	48.00–1.93 (1.96–1.93)	47.59–2.45 (2.55–2.45) ^a^	45.31–1.42 (1.44–1.42)
*R*_sym_ or *R*_merge_	0.145 (1.007)	0.185 (1.040)	0.068 (0.788)	0.108 (1.473)	0.104 (0.792)	0.075 (1.341)
*I* / σ*I*	8.62 (2)	10.9 (1.9)	14.3(1.9)	13.6 (2.0)	9.4 (1.9)	18.0 (2.0)
Completeness (%)	100.0 (100.0)	99.9 (100.0)	98.9 (99.8)	100.0 (99.9)	96.7 (98.8)	100.0 (100.0)
Redundancy	7.5 (7.2)	7.3 (6.8)	6.6 (6.4)	13.2 (13.4)	4.8 (5.0)	12.7 (10.0)
**Refinement**						
Resolution (Å)	47.57–2.00 (2.07–2.00)	44.83 – 2.28 (2.37–2.28)	37.60–1.75 (1.81–1.75)	48.00–1.87 (1.99–1.93)	38.94–2.45 (2.53– 2.45)	42.98–1.42 (1.47–1.42)
No. reflections	42,627 (4,175)	45,340 (3,597)	62,159 (6,182)	94,685 (9,326)	22,491 (2,265)	116,775 (11,530)
*R*_work_ / *R*_free_	0.1809/0.2135	0.2007/0.2317	0.1785/0.2113	0.1816/0.2078	0.1904/0.2295	0.1811/ 0.1967
No. atoms	4,566	7,293	4,671	8,582	4,163	4,726
Protein	4,250	6,922	4,268	7,960	4,083	4,286
Ligand/ion	48	0	16	28	4	11
Water	268	371	387	594	18	429
*B*-factors	35.53	38.31	19.66	33.36	54.66	27.41
Protein	35.24	38.19	18.40	32.84	54.68	26.50
Ligand/ion	47.63		21.36	44.27	83.49	30.17
Water	37.96	40.47	30.59	39.86	43.6	36.41
R.m.s. deviations						
Bond lengths (Å)	0.008	0.002	0.007	0.008	0.002	0.006
Bond angles (°)	0.93	0.64	1.02	1.04	0.55	1.10

^a^ Values in parentheses are for highest-resolution shell.

For the AstD dataset, collection took place at Beamline BL18U1 at SSRF, with diffraction at a wavelength of 0.97930 Å. The best crystals reached a resolution of 2.30 Å. Indexing, integration, and scaling were performed using the HKL2000 package, and the crystals were categorized under space group P212121, as summarized in Table 1.

The *Ath*DHAD–V496W complex data were gathered at 100 K from Beam Line 18U1 at SSRF, with a diffraction wavelength of 0.97930 Å. The best crystals achieved a resolution of 1.75 Å. The Ramachandran plot indicated 98.23% of residues in favored regions, 1.77% in allowed regions, and none in outlier regions. The HKL3000 package [18] was used for data processing. These crystals belonged to space group P42212, with statistics detailed in Table 1.

Data for the *Ath*DHAD–V497F complex were collected at 100 K using Beam Line 19U1 at SSRF, with a diffraction wavelength of 0.97892 Å. The highest-quality crystals diffracted to a resolution of 1.93 Å. The Ramachandran plot showed 98.36% of residues in favored regions, 1.64% in allowed regions, and none in outlier regions. Data processing was performed using the XDS package [17], and the crystals were assigned to space group P41212, with summarized data in Table 1.

For the *Ath*DHAD–I177F complex, data collection occurred at 100 K at Beam Line 19U1 at SSRF, with a diffraction wavelength of 0.97853 Å. The best crystals provided a resolution of 2.45 Å. The Ramachandran plot showed 96.87% favored regions, 2.95% allowed regions, and 0.18% outlier regions. Indexing, integration, and scaling were done using the XDS package [17], and the crystals were classified under space group P41212, with details in Table 1.

The *Ath*DHAD–V178W complex data were collected at 100 K from Beam Line 17U1 at SSRF, with a diffraction wavelength of 0.97919 Å. The best crystals reached a resolution of 1.42 Å. According to the Ramachandran plot, 98.23% of residues were in favored regions, 1.59% in allowed regions, and 0.18% in outlier regions. Data processing was conducted using the XDS package [17], and the crystals were assigned to space group P41212, as detailed in Table 1.

### Structure determination and refinement

The structure was solved by the molecular replacement method Phaser embedded in the Phenix suite [19] and the *holo-*DHAD structure (PDB_ID: 5YM0) as the search model. The model was built in Coot, and the final refinement was done by the phenix. refine program of Phenix suite [20]. The detailed refinement statistics are summarized in Table 1. The geometry of the model was validated by Coot [21]. The picture is drawn by Pymol software [22].

### Homology modeling

We used Visual Molecular Dynamics (VMD) to align *holo*-*Ath*DHAD with *apo*-AstD to construct residues 191 to 241, [2Fe–2S] cluster, and Mg^2+^.

### In vitro bioactivity assays of *Ath*DHAD and mutants

In vitro activity assays were carried out in 25 μl of reaction mixture containing A10 buffer without NaCl. The Michaelis–Menten constants (*k*_*cat*_, *K*_M_) for each enzyme were calculated by fitting the following formula to the observed reaction rate.

$$V=\frac{V_{\max}\left[S\right]}{K_{M}+\left[S\right]}$$

$$V_{\max}=k_{\mathrm{cat}}E_{0}$$

The inhibition percentages of AA on *Ath*DHADs were determined using in vitro biochemical assays and calculated with the following equation:

Inhibition percentage = 100 − 100 × (initial reaction rate with AA/initial reaction rate without AA)

The inhibition assays were conducted with either 10 mM (±)-sodium-α,β-dihydroxyisovalerate (DHI) or 10 mM (±)-sodium-α,β-dihydroxymethylvalerate hydrate (DHMV), along with 0.5 μM of purified *Ath*DHAD enzyme or its mutants. The reaction was initiated by adding the corresponding enzymes, followed by a 20-min incubation at 28°C. To halt the reactions, an equal volume of acetonitrile was introduced. Subsequently, approximately 0.04 volumes of 100 mM phenylhydrazine were added to facilitate the derivatization of the product, either 2-keto-isovalerate or 2-keto-3-methylvalerate, at room temperature for 30 min. For HPLC analysis, 10 μl of the reaction mixture was utilized. The area under the HPLC peak exhibiting ultraviolet absorption at 341 nm was employed to quantify the amount of product formed.

The product concentration was calculated using the calibration curve depicted in Fig. S25.

### Construction of the *Ath*DHAD V178L and V496L transgenic plants

The gene block containing DHAD variants, and the 2XFLAG-tag was synthesized and then cloned into modified pEG302 vector using Gateway LR Clonase II Enzyme Mix (Thermo Fisher Scientific). Ubiquitin-10 promoter was used to drive the expression of DHAD variants. The construct was electrotransformed into *Agrobacterium tumefaciens* strain Agl0 and then transformed into *A. thaliana rdr6-15* in which the transgene silencing pathway is blocked. Positive transgenic plants were selected using the hygromycin B and were tested for survival in the presence of AA.

### Western blot

About 100 mg of leaf tissue of transgenic plants was ground in liquid nitrogen, and then 100 μl of 2XSDS buffer was added for protein extraction. The protein samples were loaded onto a 4% to 12% Bris-tris gel and separated using Mops running buffer. Proteins were then transferred to the polyvinylidene fluoride membrane for antibody inoculation, with detection using the Amersham ECL Prime detection reagent. The anti-FLAG M2-Perocidase antibody was from Sigma (A8592-1MG). The total protein was stained with Ponceau S to demonstrate equal loading.

### Growth inhibition of AA on *A. thaliana*

*A. thaliana* seeds were sterilized with 70% ethanol and sown on Murashige and Skoog medium supplemented with either ethanol control or 100 μM AA. Photos were taken, and fresh weights were measured at day 7.

### Statistical analysis

One-way analysis of variance followed by Tukey’s post hoc test was performed. The Scheffé multiple comparison was applied for testing correction. Statistical significance was indicated with different letters. *P* values and sample numbers (*n*) were detailed in figure legends.

## Results

### The complex structure of *Ath*DHAD and AA complex

In our previous work, we obtained the intact crystal structure of *Ath*DHAD through chemically reconstructing the 2Fe–2S cluster under anaerobic condition. We modified this process by synthesizing iron–sulfur cluster in *E. coli* by coexpressing the plasmid PDB1281 [23,24]*. Ath*DHAD was purified and mixed with AA in an anaerobic box, as well as screened for crystallization. The complex structure was solved by molecular replacement using the structure of *Ath*DHAD as a searching model and refined to resolution of 2.0 Å (Table 1). In the dimer structure of *Ath*DHAD and AA complex, the active site is located at the interspace between the N-terminal domain (residues 1 to 388) and the C-terminal domain (residues 394 to 573), formed by 2Fe–2S cluster and Mg^2+^ (Fig. 1B and Fig. S1A and B). The 2Fe–2S cluster is coordinated with C66, C139, and C211, with an occupancy of 0.7 in the active sites (Fig. S1B and C). The Mg^2+^ in the active site is 6.45 Å away from the 2Fe–2S cluster and coordinated with D140, D98, and E463 (Fig. 1D). The substrate channel consists of 2 α-helices (residues 177 to 198 and 465 to 474) and 5 loops (residues 99 to 102, 211 to 215, 324 to 327, 409 to 412, and 489 to 493) (Fig. S3). AA was observed at the active site and the substrate channel with an occupancy of 0.90 (Fig. 1B and Fig. S1D). AA fills essentially the entire substrate pocket via polar and hydrophobic interactions (Fig. 1C). The carboxyl and hydroxyl groups of AA are coordinated with Mg^2+^. The carboxyl oxygen atoms of AA are within hydrogen bonding distance (<3.5 Å) with the backbone nitrogen atoms and side-chain oxygen atoms of T216, carboxyl oxygen atom of E463, carboxyl oxygen atom of D140, and a water (**W**) which is located 2.75 Å away from the iron atom of 2Fe–2S cluster. The (2*R*)-α-hydroxyl of AA is within hydrogen bonding distance (<3.5 Å) to the carboxylates of D140 and E463 and is within coordination distance to the Mg^2+^ as well. The β-ether oxygen of AA participates in the formation of hydrogen bond networks via **W**. Hence, the closely positioned [2Fe–2S], Mg^2+^, **W**, and the residues T216, E463, and D140 all contribute to anchoring the hydrophilic side of AA in the active site. These hydrogen bonds and coordination interactions may mimic the binding mode of the (2*R*, *3R*)-dihydroxy and carboxyl groups in natural substrates such as DHI [25] and DHMV (Fig. 1A). The **W** molecule could represent the binding mode of the protonated hydroxide byproduct of the β-dehydration reaction catalyzed by DHAD, in which the [2Fe–2S] cluster is proposed to play a role as a Lewis acid.

### The conformational change of the activesite when AA binding

AA is considerably larger compared to the natural substrate of DHAD due to the nonpolar 5,7-bicyclic ring system. Extensive hydrophobic interactions between the nonpolar regions of AA and residues that line the substrate entry channel, including M102, I177, V178, F181, Y215, and L465, are observed in the complex structure (Fig. 1C). Such nonpolar interactions are not possible with either DHI or DHMV and rationalize the strong binding of AA to *Ath*DHAD (*K*_i_ ~ 300 nM) as a competitive inhibitor [15]. Although the overall structure of *Ath*DHAD and *Ath*DHAD–AA complex are almost identical, with a root-mean-square deviation (RMSD) of 0.171 Å for 1,002 core C_α_ atoms. Binding of AA expanded the active chamber by pushing outward the residues forming the cavity, including residues I177, V178, F181, G490, and E463 (Fig. S2C). For example, to accommodate the isobutylene side chain of AA, the benzene ring of F181 rotates away about 50°, while V178 shifts ~1 Å away. This results in shifting of α-helix (formed by residues 178 to 200) ~0.6 Å away (Fig. S2A). In addition, binding of AA induces a 28° rotation of Y215 to be closer the cyclopentane of AA. The distance between V178 and G490, 2 residues forming the narrowest part of the active site, is 8.0 Å in *Ath*DHAD (Fig. S4B), whereas the corresponding distance extends to 8.6 Å when AA is bound (Fig. S4A). The binding of AA moves Mg^2+^ about 0.7 Å deeper into the pocket and changes the side chain of coordinating residues. The distance between the coordinating residue E463 and Mg^2+^ gets longer from 2.5 to 3.1 Å, whereas the side chain of D98 rotates toward the Mg^2+^ and the distance changes from 3.6 to 2.6 Å. The distance between D140 and Mg^2+^ is still 2.2 Å, although the side chain of D140 rotates (Fig. S2B). The *Ath*DHAD–AA complex structure reveals the binding mode of AA and further underscores the remarkably efficient biosynthetic logic used by Nature to craft a potent natural product inhibitor.

### The crystal structure of AstD

**Fig. 2.**
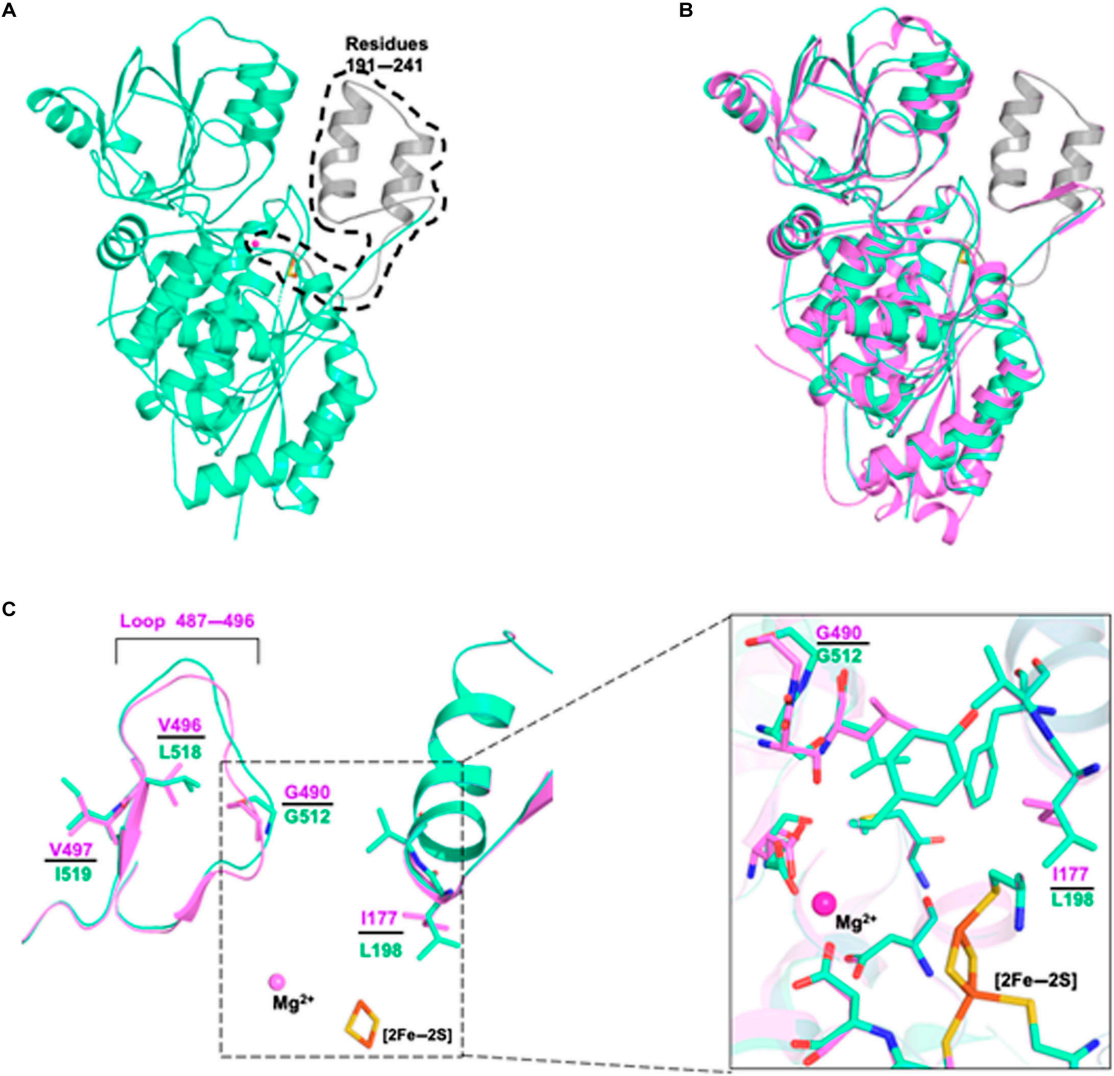
Comparison of AstD with *Ath*DHAD. (A) The monomer of AstD. The green-cyan part is crystal structure. The residues 191 to 241 are in gray, and [2Fe–2S] cluster and Mg^2+^ are molded in by VMD [26]. (B) The alignment of AstD (green-cyan) to *Ath*DHAD (violet). The RMSD is 0.706 Å (462 to 462 Cα atoms). (C) The difference of *Ath*DHAD and AstD at the loop 487–496 caused by the difference of V497I and V496L. The superimposing of the active site of *Ath*DHAD (violet) and AstD (green-cyan).

The SRE enzyme AstD shares high sequence identity (61.3% with 91% coverage) with *Ath*DHAD and catalyzes the identical β-dehydration reaction as *Ath*DHAD, albeit with a more than 20-fold reduction in *k*_*cat*_. AstD, however, is completely insensitive to AA within the solubility limit (8 mM). To understand the resistance mechanism, we determined its crystal structure (2.30 Å) by molecular replacement using the *Ath*DHAD as a searching model. Although AstD was purified and crystallized under the same anaerobic condition as that of *Ath*DHAD, the electron density of [2Fe–2S] cluster, Mg^2+^, and residues 191 to 241 cannot be observed in the crystal structure (Fig. 2A). The loss of [2Fe–2S] in recombinant DHAD in the absence of a bound ligand has been reported in other DHAD and homologous enzymes and leads to loss of well-defined structures in the region noted [25].

We generated homology models of this missing region, along with Mg^2+^ and [2Fe–2S] cluster with VMD [26], using the structure of *Ath*DHAD as a template. The overall structures of AstD and *Ath*DHAD are very similar, with an RMSD of 0.706 Å for 462 backbone C_α_ atoms (Fig. 2B). Residues E485, D160, and D118 that coordinated with Mg^2+^, residues C86, C159, and C232 that coordinated with [2Fe–2S], and other residues in the active chamber are all well conserved as would be expected for a functional DHAD (Fig. 2C). The main difference around the AA binding sites is the size of the substrate channel. The distance between V199 and G512 in AstD, the conserved residues forming the narrowest part of the active site entrance, is 7.7 Å, whereas the corresponding distance in *Ath*DHAD is 8.0 Å (Fig. S4B and C). Overlay of the structures showed that 3 amino acid differences (V496L, V497I, and I177L) between the 2 enzymes may lead to a more constricted entrance (Fig. 2C). We reasoned that narrowing the entrance, along with other mutations away from the active site, may prevent the bulky AA from entering in the active site. The substitutions of V496L and V497I may push the loop where G490 is located toward the center of the channel. The substitution of I177L might result in smaller active cavity.

### The self-resistance mechanism of AstD

To determine if small-to-large amino acid mutations at these hydrophobic residues can decrease active site volume and increase resistance to AA, we generated multiple mutations at V496, V497, and I177 in *Ath*DHAD followed by kinetic and structural characterizations. Furthermore, we also generated mutants of V178, which was shown to interact with the isopropylcyclopentane portion of AA in the *Ath*DHAD–AA complex structure (Fig. S5). Both DHI and DHMV were used as substrates for calculating the kinetic parameters of mutants. The half-maximal inhibition concentration (IC_50_) of AA on *Ath*DHAD and its mutants were measured using DHI as substrate using different concentrations of AA (Figs. S6 to S21). The α-ketoacid products from the reaction were derivatized with phenylhydrazine for ultraviolet detection at λ = 341 nm (Fig. S4), and the concentrations were quantified with a calibration curve depicted in Fig. S22. Parameters representing enzymatic activities of *Ath*DHAD and its mutants are summarized in Table 2 and Table S3. When DHI served as the substrate, the Michaelis–Menten constants *k*_*cat*_ and *K*_M_ of *Ath*DHAD were 6.85 ± 0.51 s^−1^ and 5.78 ± 1.06 mM, respectively (Table S3). Notably, the *k*_*cat*,_ value for DHI exhibited an increase compared to the previously reported value of 1.2 s^−1^, while the *K*_M, DHI_ value remained consistent. This enhancement in activity can be attributed to the refined purification process, aimed at maximizing enzymatic efficacy. Likewise, when DHMV was utilized as the substrate, *k*_*cat*_ and *K*_M_ were determined as 6.50 ± 0.32 s^−1^ and 4.00 ± 0.45 mM, respectively. The catalytic activity exhibited similarity between the 2 substrates, aligning with expectations for the wild-type enzyme (Table S3). Additionally, the inhibitory concentration (IC_50_) for AA toward *Ath*DHAD was found to be 0.25 ± 0.00 μM, a result consistent with a previous report [15].

**Table 2.** The IC_50_ and *k*_*cat*_/*K*_M_ calculations of *Ath*DHAD and its mutants

		*k*_*cat*_ /*K*_M_ (s^−1^mM^−1^)	
Enzyme	IC_50_ (μM)	DHI	DHMV
*Ath*DHAD	0.249 ± 0.000	1.185 ± 0.189	1.622 ± 0.143
I177L	0.050 ± 0.001	0.270 ± 0.015	0.280 ± 0.014
I177F	37.000 ± 8.77	0.023 ± 0.003	0.027 ± 0.007
I177W		N/A	
V178L	0.974 ± 0.064	0.153 ± 0.015	0.154 ± 0.004
V178I	0.431 ± 0.016	1.626 ± 0.491	1.077 ± 0.136
V178F	5.991 ± 0.185	0.010 ± 0.001	0.019 ± 0.003
V178W	2.337 ± 0.027	0.009 ± 0.004	0.002 ± 0.000
V496L	0.824 ± 0.004	1.897 ± 0.233	0.983 ± 0.106
V496I	1.140 ± 0.052	0.306 ± 0.060	0.148 ± 0.031
V496F	938 ± 0.012	0.624 ± 0.216	0.249 ± 0.240
V496W		N/A	
V497L	0.059 ± 0.000	1.399 ± 0.034	1.070 ± 0.146
V497I	0.073 ± 0.001	0.882 ± 0.095	0.545 ± 0.027
V497F	1.246 ± 0.006	0.051 ± 0.006	0.036 ± 0.008
V497W		N/A	
I177L/V496L	0.309 ± 0.074	1.936 ± 0.260	0.760 ± 0.065
I177L/V497L	0.028 ± 0.000	0.107 ± 0.003	0.120 ± 0.004
V178L/V497L	0.396 ± 0.011	0.460 ± 0.018	0.642 ± 0.010

N/A, the biochemical data was not obtainable

All V496 mutants had increased AA resistance, where the IC_50_ values were 0.82, 1.14, and 2.94 μM for V496L, V496I, and V496F, respectively. The *k*_*cat*_ and *k*_*cat*_/*K*_M_ values of the V496L and V496I mutants were found not to be obviously compromised, suggesting that the side chains of these mutants were sufficiently large to enhance resistance by inwardly pushing the 489–493 loop. However, they were not excessively large to hinder the entry of native substrates into the active site. Additionally, the inhibitory concentration (IC_50_) of AA for the V497L, V497I, and V497F mutants were determined as 0.059, 0.0733, and 1.25 μM, respectively.

TI177L and I177F mutants had an IC_50_ of 0.05 and 37.0 μM. The AA resistance of all V178 mutants were increased, where the IC_50_ values were 0.97, 0.43, 6.00, and 2.34 μM for V178L, V178I, V178F, and V178W mutants, respectively. The observed increases in IC_50_ suggest that the larger hydrophobic side chains of these mutants effectively narrowed the entrance to the active site, thereby impeding the entry of AA into the catalytic region. We also combined few of the Leu mutants to evaluate if these mutations had synergistic effect on AA resistance. Unfortunately, the double mutants were not able to greatly affect the resistance (Table 2). Overall, we saw increase in resistance when these amino acids were mutated to larger hydrophobic amino acids like Leu and Ile, while the enzyme activity gets severely compromised if these mutations are overly large like Phe and Trp (Table 2 and Table S3).

**Fig. 3.**
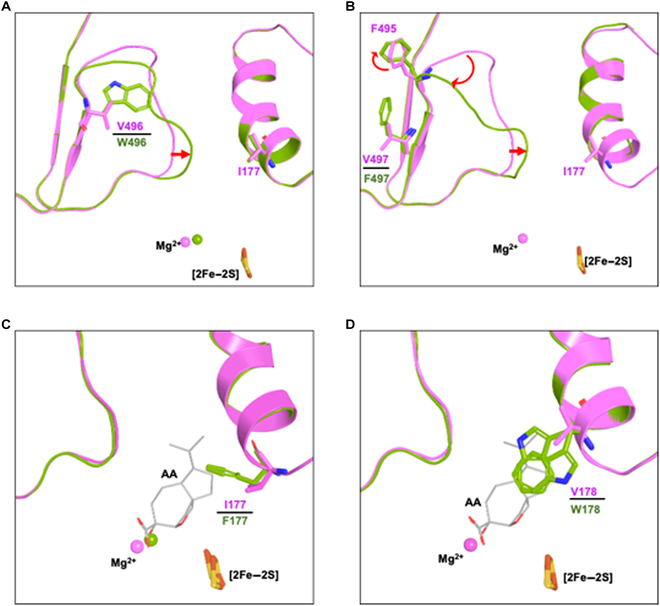
Comparison of the crystal structures of *Ath*DHAD with 4 *Ath*DHAD mutants. (A) Superimpositions of the active site in *Ath*DHAD (violet) with *Ath*DHAD–V496W (green). (B) Superimpositions of the active site in *Ath*DHAD (violet) with *At*DHAD-V497F (green). (C) Superimpositions of the active site in *Ath*DHAD (violet) with *Ath*DHAD–I177F (green). The virtual location of AA is in gray. (D) Superimpositions of the active site in *Ath*DHAD (violet with *At*DHAD-V178W (green). The virtual location of AA is in gray.

To probe the acquired resistance mechanism of *Ath*DHAD mutants, we determined the crystal of *Ath*DHAD–I177F (2.45 Å), *Ath*DHAD–V178W (1.77 Å), *Ath*DHAD–V496W (1.59 Å), and *Ath*DHAD–V497F (1.87 Å). In the crystal structures of I177F, V178W, V496W, and V497F, the occupancy of [2Fe–2S] cluster is 0.64, 0.72, 0.63, and 0.77, respectively. The overall structures of the *Ath*DHAD mutants I177F, V178W, V496W, and V497F displayed high similarity with the wild-type *Ath*DHAD, with the RMSD of 0.123 Å (888 core C_α_ atoms), 0.275 Å (994 core C_α_ atoms), 0.142 Å (1,034 core C_α_ atoms), and 0.283 Å (994 core C_α_ atoms), respectively (Fig. S5A to D). The obvious difference was in loop 490–497, which forms the side wall at the narrowest point of the substrate channel (Fig. 2C). For example, in the V496W structure, the side chain of W496 induced the loop 489–493 move toward V178, which made the chamber entrance narrower and might block AA to access the active site (Fig. 3A). In the V497F structure, the benzene ring of F497 induces F495 to rotate about 53° which change the shape of loop 489–493 and make it close to the V178 at the channel entrance. As a result, this mutant also affects the accession of AA to the active site (Fig. 3B). Both V178 and I177 are close to the active site. I177F make the cavity smaller, so that the cavity cannot accommodate the hydrophobic part of AA (Fig. 3C). Alignment of the *Ath*DHAD–AA complex structure to V178W reveals that the side chain of W178 has 2 conformations and not only makes the channel entrance narrower but also occupies the position of isopropylcyclopentane of AA (Fig. 3D).

We also investigated if some point mutants of *Ath*DHAD can have resistance effects toward AA *in planta*. Two *A. thaliana* transgenic lines with mutant *Ath*DHAD (V178L and V496L) were generated through *Agrobacterium tumefaciens* and were grown in the presence of lethal dose of AA. However, compared to the transgenic *A. thaliana* line expressing AstD, these mutants were not able to rescue the growth of the plants when exposed to 100 μM AA (Fig. S26), suggesting further mutations to improve resistance toward AA is required.

## Discussion

In summary, we analyzed the crystal structure of *Ath*DHAD–AA complex, which revealed the key groups of AA for inhibition. The coordination characteristics and hydrogen bonding network of hydroxycarboxylic acids, the extensive hydrophobic interactions of nonpolar 5,7-bicyclic ring system, and the just right molecular size—these will all have important reference value for the research and development of herbicides targeting DHAD. The self-resistance mechanism of AstD also supplies a potential for producing resistant crops and creating new weed management system. Our structural and kinetic analysis of the mutant *Ath*DHADs has validated the hypothesis that larger hydrophobic amino acids surrounding the active-site entrance enhance resistance to AA. However, it is evident that while these amino acids near the active-site entrance play a crucial role in AA resistance, they alone do not fully account for the remarkable high resistance observed in AstD, reported to be over 8 mM. Additionally, it is noteworthy that heightened AA resistance correlates with compromised enzyme activity. This is evident in the disparity between the *k*_*cat*_ values of *Ath*DHADs and the reported *k*_*cat*_ of *Ath*DHAD, measured at 6.85 and 0.03 s^−1^, respectively, implying that increased AA resistance coincides with diminished enzyme activity.

Although point mutations made to *Ath*DHAD, based on structural comparison of *Ath*DHAD and AstD, were not able to increase its AA resistance to the high level of AstD, they give insights into how the active site entrance affects the activity and resistance of the enzyme.

To gain a comprehensive understanding of the additional mutations that may contribute to resistance, it may be necessary to pursue directed evolution methods based on our previously developed yeast platform [8]. With further evolutionary and in planta screening, AA in combination with AstD or DHAD mutant is a promising lead for herbicide development.

## Data Availability

Submission of a structural factor and coordinate have been deposited in the Protein Bank. PDB IDs are 9JPI (*Ath*DHAD–AA), 9IX7 (AstD), 8IKZ (*Ath*DHAD–V496W), 8IMU (*Ath*DHAD–V497F), 9JSQ (*Ath*DHAD–I199F), and 8HS0 (*Ath*DHAD–V178W).
