# Supplementary material for: Structural Bases of Dihydroxy Acid Dehydratase Inhibition and Biodesign for Self-Resistance
Source: Biodes Res. 2024 Nov 1;6:0046. doi: 10.34133/bdr.0046 (PMC11528067; doi:10.34133/bdr.0046)
Supplement: Supplementary 1 — Tables S1 to S7 Figs. S1 to S26 [file bdr.0046.f1.zip › D_1300031929_val-report-full_P1.pdf]

Full wwPDB X-ray Structure Validation Report (i)

Mar 16, 2023 – 11:39 AM JST

PDB ID	:	8GRC
Title	:	The mutant structure of Dihydroxyacid dehydratase (DHAD)-I177F
Deposited on	:	2022-09-01
Resolution	:	2.45 Å(reported)

This wwPDB validation report is for manuscript review

This is a Full wwPDB X-ray Structure Validation Report.

This report is produced by the wwPDB biocuration pipeline after annotation of the structure.

We welcome your comments at *validation@mail.wwpdb.org*
A user guide is available at
https://www.wwpdb.org/validation/2017/XrayValidationReportHelp
with specific help available everywhere you see the (i) symbol.

The types of validation reports are described at
http://www.wwpdb.org/validation/2017/FAQs#types.

For Manuscript Review

The following versions of software and data (see references (i)) were used in the production of this report:

MolProbity	:	4.02b-467
Mogul	:	1.8.5 (274361), CSD as541be (2020)
Xtriage (Phenix)	:	1.13
EDS	:	2.32.1
buster-report	:	1.1.7 (2018)
Percentile statistics	:	20191225.v01 (using entries in the PDB archive December 25th 2019)
Refmac	:	5.8.0158
CCP4	:	7.0.044 (Gargrove)
Ideal geometry (proteins)	:	Engh & Huber (2001)
Ideal geometry (DNA, RNA)	:	Parkinson et al. (1996)

1 Overall quality at a glance (i)

The following experimental techniques were used to determine the structure:
X-RAY DIFFRACTION

The reported resolution of this entry is 2.45 Å.

Percentile scores (ranging between 0-100) for global validation metrics of the entry are shown in the following graphic. The table shows the number of entries on which the scores are based.

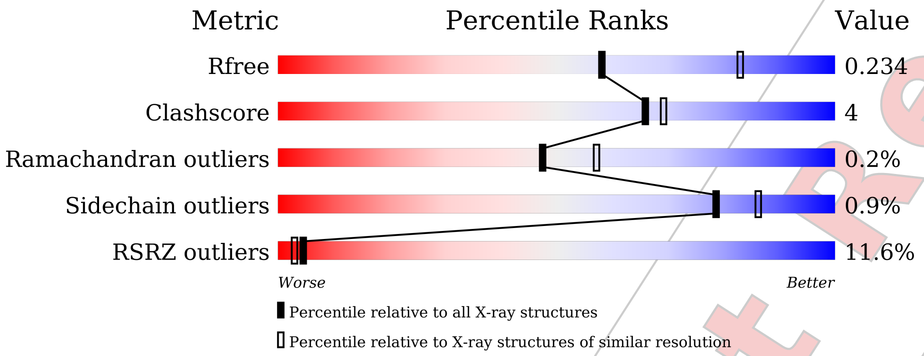

Metric	Whole archive (#Entries)	Similar resolution (#Entries, resolution range(Å))
R_{free}	130704	1544 (2.48-2.44)
Clashscore	141614	1613 (2.48-2.44)
Ramachandran outliers	138981	1598 (2.48-2.44)
Sidechain outliers	138945	1598 (2.48-2.44)
RSRZ outliers	127900	1523 (2.48-2.44)

The table below summarises the geometric issues observed across the polymeric chains and their fit to the electron density. The red, orange, yellow and green segments of the lower bar indicate the fraction of residues that contain outliers for >=3, 2, 1 and 0 types of geometric quality criteria respectively. A grey segment represents the fraction of residues that are not modelled. The numeric value for each fraction is indicated below the corresponding segment, with a dot representing fractions <=5% The upper red bar (where present) indicates the fraction of residues that have poor fit to the electron density. The numeric value is given above the bar.

Mol	Chain	Length	Quality of chain
1	A	580	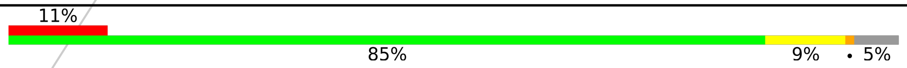

For Manuscript Review

2 Entry composition (i)

There are 3 unique types of molecules in this entry. The entry contains 4163 atoms, of which 0 are hydrogens and 0 are deuteriums.

In the tables below, the ZeroOcc column contains the number of atoms modelled with zero occupancy, the AltConf column contains the number of residues with at least one atom in alternate conformation and the Trace column contains the number of residues modelled with at most 2 atoms.

- Molecule 1 is a protein called Dihydroxy-acid dehydratase, chloroplastic.

Mol	Chain	Residues	Atoms					ZeroOcc	AltConf	Trace
			Total	C	N	O	S			
1	A	551	4141	2594	701	811	35	0	7	0

There are 4 discrepancies between the modelled and reference sequences:

Chain	Residue	Modelled	Actual	Comment	Reference
A	-5	GLY	-	expression tag	UNP Q9LIR4
A	177	PHE	ILE	engineered mutation	UNP Q9LIR4
A	525	ALA	LYS	engineered mutation	UNP Q9LIR4
A	526	ALA	LYS	engineered mutation	UNP Q9LIR4

- Molecule 2 is FE2/S2 (INORGANIC) CLUSTER (three-letter code: FES) (formula: Fe_2S_2) (labeled as "Ligand of Interest" by depositor).

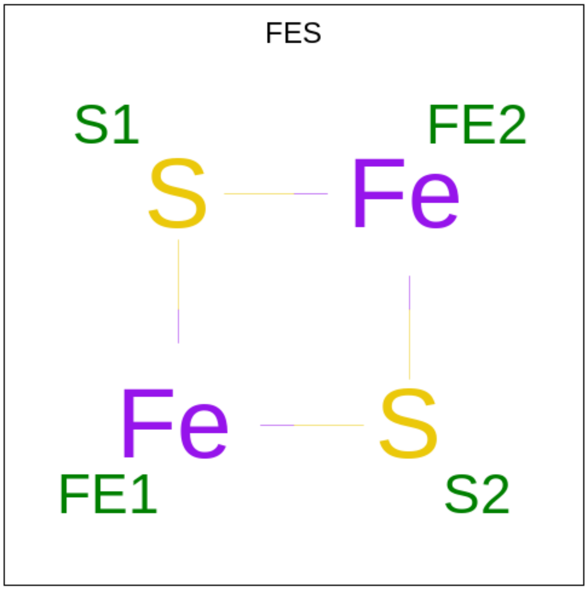

Mol	Chain	Residues	Atoms			ZeroOcc	AltConf
			Total	Fe	S		
2	A	1	4	2	2	0	0

For Manuscript Review

- Molecule 3 is water.

Mol	Chain	Residues	Atoms		ZeroOcc	AltConf
			Total	O		
3	A	18	18	18	0	0

For Manuscript Review

3 Residue-property plots (i)

These plots are drawn for all protein, RNA, DNA and oligosaccharide chains in the entry. The first graphic for a chain summarises the proportions of the various outlier classes displayed in the second graphic. The second graphic shows the sequence view annotated by issues in geometry and electron density. Residues are color-coded according to the number of geometric quality criteria for which they contain at least one outlier: green = 0, yellow = 1, orange = 2 and red = 3 or more. A red dot above a residue indicates a poor fit to the electron density (RSRZ > 2). Stretches of 2 or more consecutive residues without any outlier are shown as a green connector. Residues present in the sample, but not in the model, are shown in grey.

- Molecule 1: Dihydroxy-acid dehydratase, chloroplastic

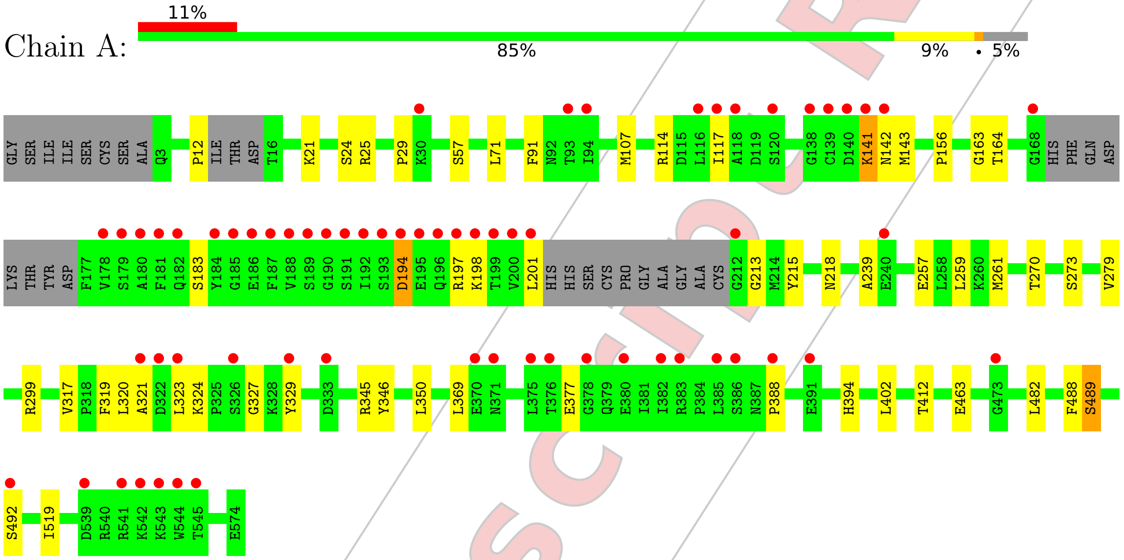

For Manuscript Review

4 Data and refinement statistics (i)

Property	Value	Source
Space group	P 42 21 2	Depositor
Cell constants<br>a, b, c, α, β, γ	135.49Å 135.49Å 66.86Å<br>90.00° 90.00° 90.00°	Depositor
Resolution (Å)	38.94 – 2.45<br>47.59 – 2.45	Depositor<br>EDS
% Data completeness<br>(in resolution range)	95.9 (38.94-2.45)<br>96.0 (47.59-2.45)	Depositor<br>EDS
R_{merge}	0.10	Depositor
R_{sym}	(Not available)	Depositor
$< I/\sigma(I) >$ [1]	1.91 (at 2.45Å)	Xtriage
Refinement program	PHENIX 1.19.2_4158	Depositor
R, R_{free}	0.198 , 0.233<br>0.198 , 0.234	Depositor<br>DCC
R_{free} test set	1125 reflections (5.00%)	wwPDB-VP
Wilson B-factor (Å^2)	46.3	Xtriage
Anisotropy	0.056	Xtriage
Bulk solvent k_{sol}(e/Å^3), B_{sol}(Å^2)	0.37 , 51.8	EDS
L-test for twinning[2]	$< \|L\| > = 0.48$, $< L^2 > = 0.31$	Xtriage
Estimated twinning fraction	No twinning to report.	Xtriage
F_o,F_c correlation	0.94	EDS
Total number of atoms	4163	wwPDB-VP
Average B, all atoms (Å^2)	54.0	wwPDB-VP

Xtriage's analysis on translational NCS is as follows: *The largest off-origin peak in the Patterson function is 4.52% of the height of the origin peak. No significant pseudotranslation is detected.*

[1] Intensities estimated from amplitudes.

[2] Theoretical values of $< |L| >$, $< L^2 >$ for acentric reflections are 0.5, 0.333 respectively for untwinned datasets, and 0.375, 0.2 for perfectly twinned datasets.

For Manuscript Review

5 Model quality (i)

5.1 Standard geometry (i)

Bond lengths and bond angles in the following residue types are not validated in this section: FES

The Z score for a bond length (or angle) is the number of standard deviations the observed value is removed from the expected value. A bond length (or angle) with $|Z| > 5$ is considered an outlier worth inspection. RMSZ is the root-mean-square of all Z scores of the bond lengths (or angles).

Mol	Chain	Bond lengths		Bond angles	
		RMSZ	#|Z| >5	RMSZ	#|Z| >5
1	A	0.24	0/4211	0.46	0/5685

There are no bond length outliers.

There are no bond angle outliers.

There are no chirality outliers.

There are no planarity outliers.

5.2 Too-close contacts (i)

In the following table, the Non-H and H(model) columns list the number of non-hydrogen atoms and hydrogen atoms in the chain respectively. The H(added) column lists the number of hydrogen atoms added and optimized by MolProbity. The Clashes column lists the number of clashes within the asymmetric unit, whereas Symm-Clashes lists symmetry-related clashes.

Mol	Chain	Non-H	H(model)	H(added)	Clashes	Symm-Clashes
1	A	4141	0	4162	33	0
2	A	4	0	0	0	0
3	A	18	0	0	0	0
All	All	4163	0	4162	33	0

The all-atom clashscore is defined as the number of clashes found per 1000 atoms (including hydrogen atoms). The all-atom clashscore for this structure is 4.

All (33) close contacts within the same asymmetric unit are listed below, sorted by their clash magnitude.

Atom-1	Atom-2	Interatomic distance (Å)	Clash overlap (Å)
1:A:213:GLY:O	1:A:218:ASN:ND2	2.33	0.59

Continued on next page...

For Manuscript Review

Continued from previous page...

Atom-1	Atom-2	Interatomic distance (Å)	Clash overlap (Å)
1:A:107:MET:HE2	1:A:463:GLU:H	1.69	0.57
1:A:114:ARG:HB2	1:A:141[A]:LYS:HB3	1.87	0.56
1:A:194:ASP:O	1:A:198:LYS:HG2	2.07	0.54
1:A:321:ALA:HB1	1:A:323:LEU:HD23	1.91	0.53
1:A:12:PRO:HG3	1:A:25:ARG:HB2	1.91	0.52
1:A:117:ILE:HD13	1:A:142[B]:ASN:HB3	1.92	0.50
1:A:183:SER:HB2	1:A:197:ARG:HG3	1.92	0.50
1:A:270:THR:H	1:A:273:SER:HG	1.60	0.49
1:A:164:THR:OG1	1:A:218:ASN:OD1	2.26	0.48
1:A:57:SER:HB3	1:A:91:PHE:CZ	2.49	0.47
1:A:319:PHE:HE2	1:A:321:ALA:HB3	1.80	0.47
1:A:323:LEU:O	1:A:327:GLY:N	2.44	0.47
1:A:345:ARG:HD2	1:A:369:LEU:O	2.15	0.47
1:A:213:GLY:H	1:A:215:TYR:HE2	1.64	0.45
1:A:71:LEU:HD23	1:A:71:LEU:HA	1.83	0.45
1:A:482:LEU:HG	1:A:488:PHE:CZ	2.51	0.45
1:A:377:GLU:H	1:A:377:GLU:CD	2.20	0.45
1:A:463:GLU:OE2	1:A:489:SER:OG	2.29	0.45
1:A:24:SER:O	1:A:29:PRO:HD3	2.16	0.45
1:A:257:GLU:O	1:A:261:MET:HG3	2.17	0.44
1:A:346:TYR:CZ	1:A:350:LEU:HD11	2.52	0.44
1:A:320:LEU:HD23	1:A:388:PRO:HB3	1.99	0.44
1:A:197:ARG:O	1:A:201:LEU:HG	2.18	0.43
1:A:324:LYS:HB3	1:A:324:LYS:HE3	1.70	0.43
1:A:163:GLY:HA3	1:A:239:ALA:HA	2.00	0.43
1:A:156:PRO:HG2	1:A:259:LEU:HD23	2.00	0.43
1:A:402:LEU:HD11	1:A:519:ILE:HG12	2.01	0.43
1:A:323:LEU:HD13	1:A:329:TYR:HB2	2.01	0.43
1:A:394:HIS:CE1	1:A:412:THR:HG22	2.54	0.42
1:A:299:ARG:HD2	1:A:299:ARG:HA	1.78	0.42
1:A:279:VAL:HG22	1:A:317:VAL:HG21	2.03	0.40
1:A:12:PRO:HD2	1:A:21:LYS:O	2.21	0.40

There are no symmetry-related clashes.

5.3 Torsion angles (i)

5.3.1 Protein backbone (i)

In the following table, the Percentiles column shows the percent Ramachandran outliers of the chain as a percentile score with respect to all X-ray entries followed by that with respect to entries

For Manuscript Review

of similar resolution.

The Analysed column shows the number of residues for which the backbone conformation was analysed, and the total number of residues.

Mol	Chain	Analysed	Favoured	Allowed	Outliers	Percentiles
1	A	550/580 (95%)	533 (97%)	16 (3%)	1 (0%)	47 57

All (1) Ramachandran outliers are listed below:

Mol	Chain	Res	Type
1	A	143	MET

5.3.2 Protein sidechains (i)

In the following table, the Percentiles column shows the percent sidechain outliers of the chain as a percentile score with respect to all X-ray entries followed by that with respect to entries of similar resolution.

The Analysed column shows the number of residues for which the sidechain conformation was analysed, and the total number of residues.

Mol	Chain	Analysed	Rotameric	Outliers	Percentiles
1	A	454/471 (96%)	449 (99%)	5 (1%)	73 82

All (5) residues with a non-rotameric sidechain are listed below:

Mol	Chain	Res	Type
1	A	141[A]	LYS
1	A	141[B]	LYS
1	A	194	ASP
1	A	489	SER
1	A	492	SER

Sometimes sidechains can be flipped to improve hydrogen bonding and reduce clashes. All (1) such sidechains are listed below:

Mol	Chain	Res	Type
1	A	531	GLN

5.3.3 RNA (i)

There are no RNA molecules in this entry.

For Manuscript Review

5.4 Non-standard residues in protein, DNA, RNA chains (i)

There are no non-standard protein/DNA/RNA residues in this entry.

5.5 Carbohydrates (i)

There are no monosaccharides in this entry.

5.6 Ligand geometry (i)

1 ligand is modelled in this entry.

In the following table, the Counts columns list the number of bonds (or angles) for which Mogul statistics could be retrieved, the number of bonds (or angles) that are observed in the model and the number of bonds (or angles) that are defined in the Chemical Component Dictionary. The Link column lists molecule types, if any, to which the group is linked. The Z score for a bond length (or angle) is the number of standard deviations the observed value is removed from the expected value. A bond length (or angle) with $|Z| > 2$ is considered an outlier worth inspection. RMSZ is the root-mean-square of all Z scores of the bond lengths (or angles).

Mol	Type	Chain	Res	Link	Bond lengths			Bond angles		
					Counts	RMSZ	$\#\|Z\| > 2$	Counts	RMSZ	$\#\|Z\| > 2$
2	FES	A	601	1	0,4,4	-	-	-		

In the following table, the Chirals column lists the number of chiral outliers, the number of chiral centers analysed, the number of these observed in the model and the number defined in the Chemical Component Dictionary. Similar counts are reported in the Torsion and Rings columns. '-' means no outliers of that kind were identified.

Mol	Type	Chain	Res	Link	Chirals	Torsions	Rings
2	FES	A	601	1	-	-	0/1/1/1

There are no bond length outliers.

There are no bond angle outliers.

There are no chirality outliers.

There are no torsion outliers.

There are no ring outliers.

No monomer is involved in short contacts.

The following is a two-dimensional graphical depiction of Mogul quality analysis of bond lengths, bond angles, torsion angles, and ring geometry for all instances of the Ligand of Interest. In addition, ligands with molecular weight > 250 and outliers as shown on the validation Tables will

For Manuscript Review

also be included. For torsion angles, if less then 5% of the Mogul distribution of torsion angles is within 10 degrees of the torsion angle in question, then that torsion angle is considered an outlier. Any bond that is central to one or more torsion angles identified as an outlier by Mogul will be highlighted in the graph. For rings, the root-mean-square deviation (RMSD) between the ring in question and similar rings identified by Mogul is calculated over all ring torsion angles. If the average RMSD is greater than 60 degrees and the minimal RMSD between the ring in question and any Mogul-identified rings is also greater than 60 degrees, then that ring is considered an outlier. The outliers are highlighted in purple. The color gray indicates Mogul did not find sufficient equivalents in the CSD to analyse the geometry.

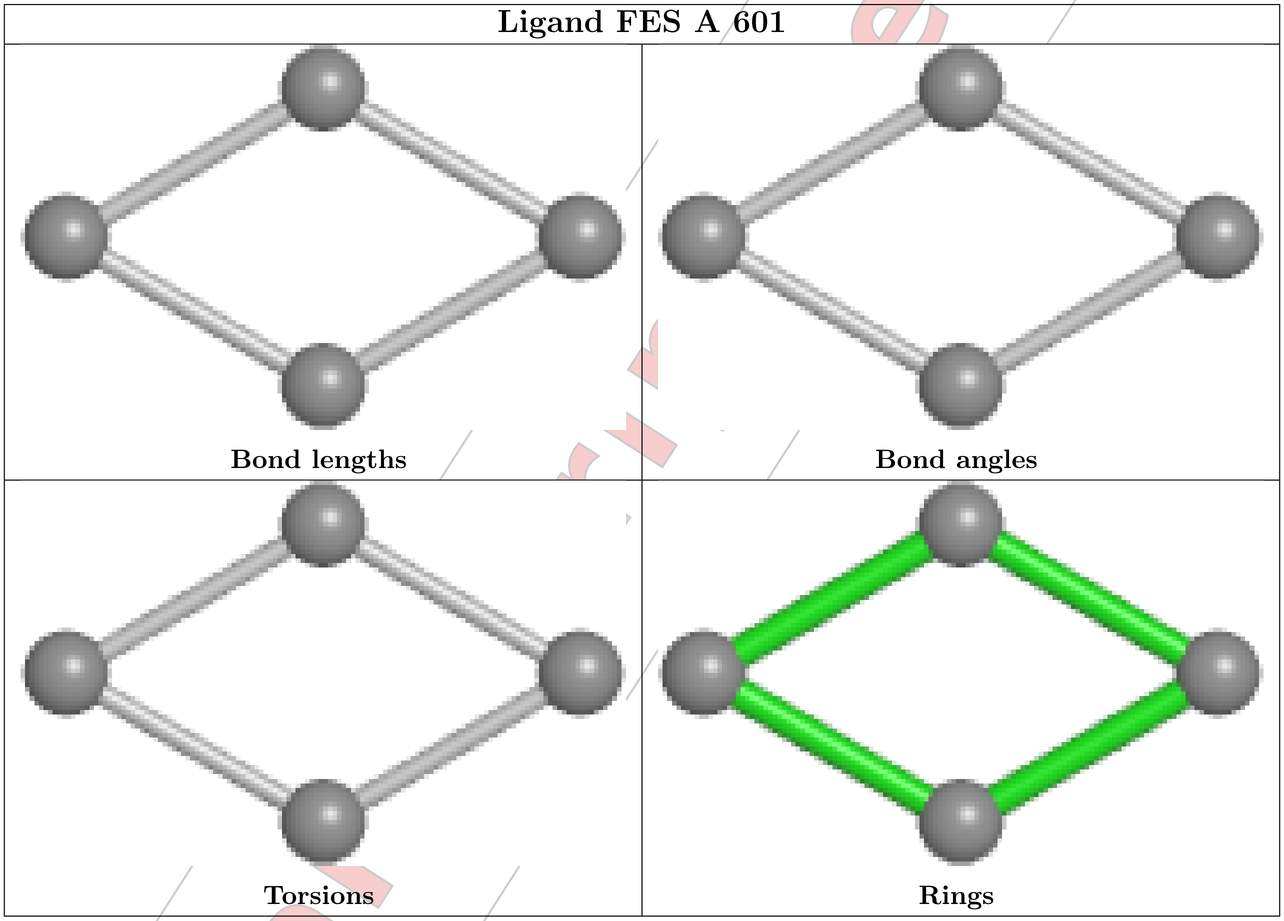

5.7 Other polymers (i)

There are no such residues in this entry.

5.8 Polymer linkage issues (i)

There are no chain breaks in this entry.

For Manuscript Review

6 Fit of model and data (i)

6.1 Protein, DNA and RNA chains (i)

In the following table, the column labelled '#RSRZ> 2' contains the number (and percentage) of RSRZ outliers, followed by percent RSRZ outliers for the chain as percentile scores relative to all X-ray entries and entries of similar resolution. The OWAB column contains the minimum, median, 95th percentile and maximum values of the occupancy-weighted average B-factor per residue. The column labelled 'Q< 0.9' lists the number of (and percentage) of residues with an average occupancy less than 0.9.

Mol	Chain	Analysed	<RSRZ>	#RSRZ>2			OWAB(Å^2)	Q<0.9
1	A	551/580 (95%)	0.43	64 (11%)	4	3	31, 48, 95, 155	1 (0%)

All (64) RSRZ outliers are listed below:

Mol	Chain	Res	Type	RSRZ
1	A	189	SER	10.2
1	A	192	ILE	6.7
1	A	187	PHE	6.5
1	A	190	GLY	6.1
1	A	191	SER	5.8
1	A	196	GLN	5.6
1	A	198	LYS	5.4
1	A	197	ARG	4.8
1	A	385	LEU	4.2
1	A	212	GLY	3.9
1	A	195	GLU	3.9
1	A	380	GLU	3.8
1	A	179	SER	3.8
1	A	329	TYR	3.7
1	A	186	GLU	3.7
1	A	382	ILE	3.7
1	A	180	ALA	3.5
1	A	326	SER	3.5
1	A	178	VAL	3.4
1	A	185	GLY	3.4
1	A	383	ARG	3.4
1	A	376	THR	3.3
1	A	199	THR	3.2
1	A	181	PHE	3.1
1	A	543	LYS	3.0
1	A	545	THR	3.0
1	A	194	ASP	3.0

Continued on next page...

For Manuscript Review

Continued from previous page...

Mol	Chain	Res	Type	RSRZ
1	A	188	VAL	3.0
1	A	544	TRP	2.9
1	A	182	GLN	2.9
1	A	139[A]	CYS	2.9
1	A	200	VAL	2.8
1	A	30	LYS	2.7
1	A	542	LYS	2.7
1	A	93	THR	2.7
1	A	492	SER	2.7
1	A	193	SER	2.7
1	A	201	LEU	2.6
1	A	371	ASN	2.6
1	A	391	GLU	2.5
1	A	375	LEU	2.5
1	A	168	GLY	2.5
1	A	321	ALA	2.5
1	A	541	ARG	2.4
1	A	117	ILE	2.4
1	A	116	LEU	2.4
1	A	323	LEU	2.4
1	A	473	GLY	2.4
1	A	142[A]	ASN	2.3
1	A	388	PRO	2.3
1	A	141[A]	LYS	2.3
1	A	94	ILE	2.3
1	A	378	GLY	2.3
1	A	370	GLU	2.3
1	A	184	TYR	2.3
1	A	333	ASP	2.2
1	A	386	SER	2.2
1	A	138[A]	GLY	2.2
1	A	539	ASP	2.2
1	A	240	GLU	2.1
1	A	322	ASP	2.1
1	A	118	ALA	2.0
1	A	120	SER	2.0
1	A	140[A]	ASP	2.0

6.2 Non-standard residues in protein, DNA, RNA chains (i)

There are no non-standard protein/DNA/RNA residues in this entry.

For Manuscript Review

6.3 Carbohydrates (i)

There are no monosaccharides in this entry.

6.4 Ligands (i)

In the following table, the Atoms column lists the number of modelled atoms in the group and the number defined in the chemical component dictionary. The B-factors column lists the minimum, median, 95th percentile and maximum values of B factors of atoms in the group. The column labelled 'Q< 0.9' lists the number of atoms with occupancy less than 0.9.

Mol	Type	Chain	Res	Atoms	RSCC	RSR	B-factors($Å^2$)	Q<0.9
2	FES	A	601	4/4	0.82	0.18	56,90,91,95	4

The following is a graphical depiction of the model fit to experimental electron density of all instances of the Ligand of Interest. In addition, ligands with molecular weight > 250 and outliers as shown on the geometry validation Tables will also be included. Each fit is shown from different orientation to approximate a three-dimensional view.

For Manuscript Review

Electron density around FES A 601:

$2mF_o$-DF_c (at 0.7 rmsd) in gray
mF_o-DF_c (at 3 rmsd) in purple (negative)
and green (positive)

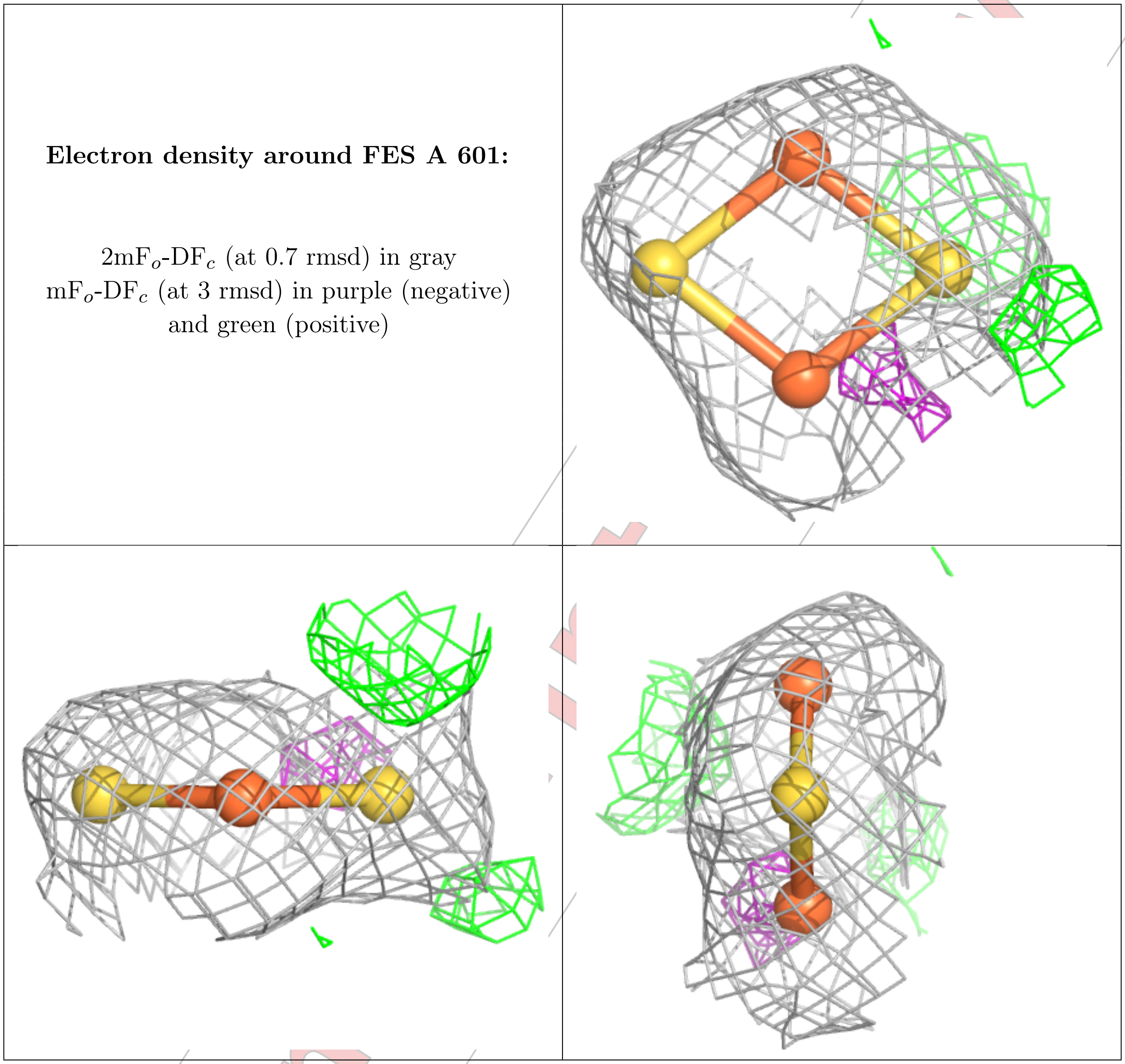

6.5 Other polymers

There are no such residues in this entry.

For Manuscript Review
