# Supplementary material for: Structural Bases of Dihydroxy Acid Dehydratase Inhibition and Biodesign for Self-Resistance
Source: Biodes Res. 2024 Nov 1;6:0046. doi: 10.34133/bdr.0046 (PMC11528067; doi:10.34133/bdr.0046)
Supplement: Supplementary 1 — Tables S1 to S7 Figs. S1 to S26 [file bdr.0046.f1.zip › D_1300035923_val-report-full_P1.pdf]

Full wwPDB X-ray Structure Validation Report (i)

Mar 9, 2023 – 01:08 PM JST

PDB ID	:	8IMU
Title	:	Dihydroxyacid dehydratase (DHAD) mutant-V497F
Deposited on	:	2023-03-07
Resolution	:	1.93 Å(reported)

This wwPDB validation report is for manuscript review

This is a Full wwPDB X-ray Structure Validation Report.

This report is produced by the wwPDB biocuration pipeline after annotation of the structure.

We welcome your comments at *validation@mail.wwpdb.org*
A user guide is available at
https://www.wwpdb.org/validation/2017/XrayValidationReportHelp
with specific help available everywhere you see the (i) symbol.

The types of validation reports are described at
http://www.wwpdb.org/validation/2017/FAQs#types.

For Manuscript Review

The following versions of software and data (see references (i)) were used in the production of this report:

MolProbity	:	4.02b-467
Mogul	:	1.8.5 (274361), CSD as541be (2020)
Xtriage (Phenix)	:	1.13
EDS	:	2.32.1
buster-report	:	1.1.7 (2018)
Percentile statistics	:	20191225.v01 (using entries in the PDB archive December 25th 2019)
Refmac	:	5.8.0158
CCP4	:	7.0.044 (Gargrove)
Ideal geometry (proteins)	:	Engh & Huber (2001)
Ideal geometry (DNA, RNA)	:	Parkinson et al. (1996)

1 Overall quality at a glance (i)

The following experimental techniques were used to determine the structure:
X-RAY DIFFRACTION

The reported resolution of this entry is 1.93 Å.

Percentile scores (ranging between 0-100) for global validation metrics of the entry are shown in the following graphic. The table shows the number of entries on which the scores are based.

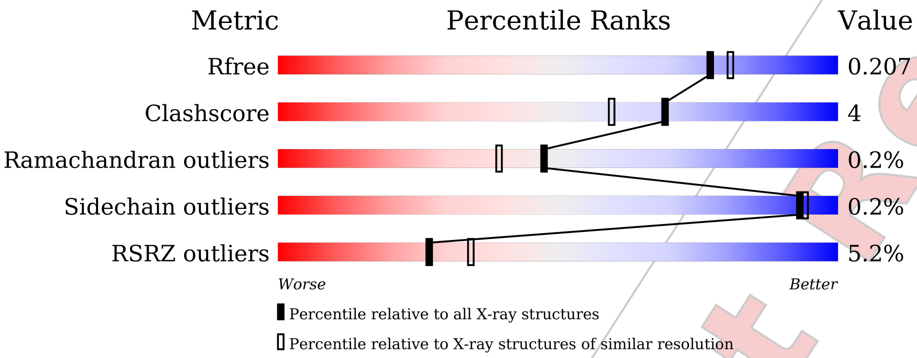

Metric	Whole archive (#Entries)	Similar resolution (#Entries, resolution range(Å))
R_{free}	130704	4310 (1.96-1.92)
Clashscore	141614	1023 (1.94-1.94)
Ramachandran outliers	138981	1007 (1.94-1.94)
Sidechain outliers	138945	1007 (1.94-1.94)
RSRZ outliers	127900	4250 (1.96-1.92)

The table below summarises the geometric issues observed across the polymeric chains and their fit to the electron density. The red, orange, yellow and green segments of the lower bar indicate the fraction of residues that contain outliers for >=3, 2, 1 and 0 types of geometric quality criteria respectively. A grey segment represents the fraction of residues that are not modelled. The numeric value for each fraction is indicated below the corresponding segment, with a dot representing fractions <=5% The upper red bar (where present) indicates the fraction of residues that have poor fit to the electron density. The numeric value is given above the bar.

Mol	Chain	Length	Quality of chain
1	A	574	5%<br>84% 7% 9%
1	B	574	5%<br>86% 5% 9%

The following table lists non-polymeric compounds, carbohydrate monomers and non-standard residues in protein, DNA, RNA chains that are outliers for geometric or electron-density-fit criteria:

For Manuscript Review

Mol	Type	Chain	Res	Chirality	Geometry	Clashes	Electron density
2	FES	B	601	-	-	X	-

For Manuscript Review

2 Entry composition (i)

There are 5 unique types of molecules in this entry. The entry contains 8582 atoms, of which 0 are hydrogens and 0 are deuteriums.

In the tables below, the ZeroOcc column contains the number of atoms modelled with zero occupancy, the AltConf column contains the number of residues with at least one atom in alternate conformation and the Trace column contains the number of residues modelled with at most 2 atoms.

- Molecule 1 is a protein called Dihydroxy-acid dehydratase, chloroplastic.

Mol	Chain	Residues	Atoms					ZeroOcc	AltConf	Trace
1	A	523	Total 3980	C 2491	N 675	O 777	S 37	0	14	0
1	B	523	Total 3980	C 2491	N 675	O 777	S 37	0	14	0

There are 8 discrepancies between the modelled and reference sequences:

Chain	Residue	Modelled	Actual	Comment	Reference
A	1	MET	-	initiating methionine	UNP Q9LIR4
A	497	PHE	VAL	engineered mutation	UNP Q9LIR4
A	525	ALA	LYS	engineered mutation	UNP Q9LIR4
A	526	ALA	LYS	engineered mutation	UNP Q9LIR4
B	1	MET	-	initiating methionine	UNP Q9LIR4
B	497	PHE	VAL	engineered mutation	UNP Q9LIR4
B	525	ALA	LYS	engineered mutation	UNP Q9LIR4
B	526	ALA	LYS	engineered mutation	UNP Q9LIR4

- Molecule 2 is FE2/S2 (INORGANIC) CLUSTER (three-letter code: FES) (formula: Fe_2S_2) (labeled as "Ligand of Interest" by depositor).

For Manuscript Review

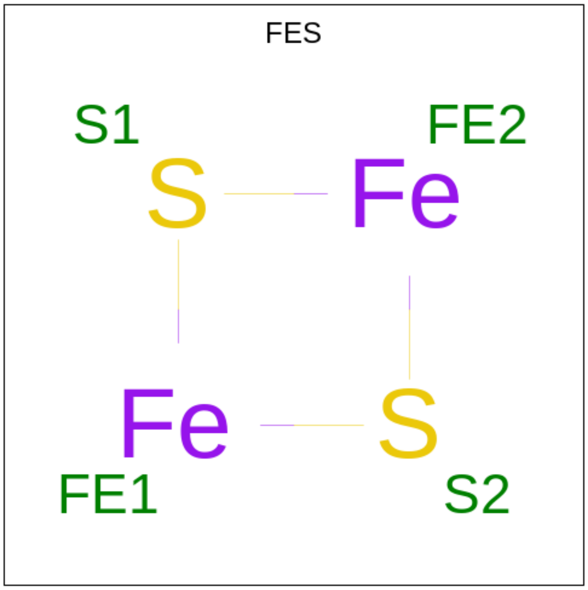

Mol	Chain	Residues	Atoms	ZeroOcc	AltConf
2	A	1	Total Fe S<br>4 2 2	0	0
2	B	1	Total Fe S<br>4 2 2	0	0

- Molecule 3 is ACETATE ION (three-letter code: ACT) (formula: $C_2H_3O_2$).

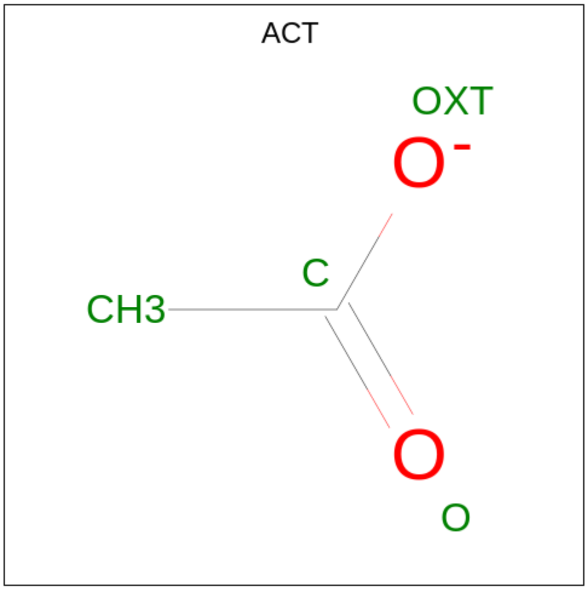

Mol	Chain	Residues	Atoms	ZeroOcc	AltConf
3	A	1	Total C O<br>4 2 2	4	0
3	B	1	Total C O<br>4 2 2	0	0

For Manuscript Review

- Molecule 4 is GLYCEROL (three-letter code: GOL) (formula: $C_3H_8O_3$).

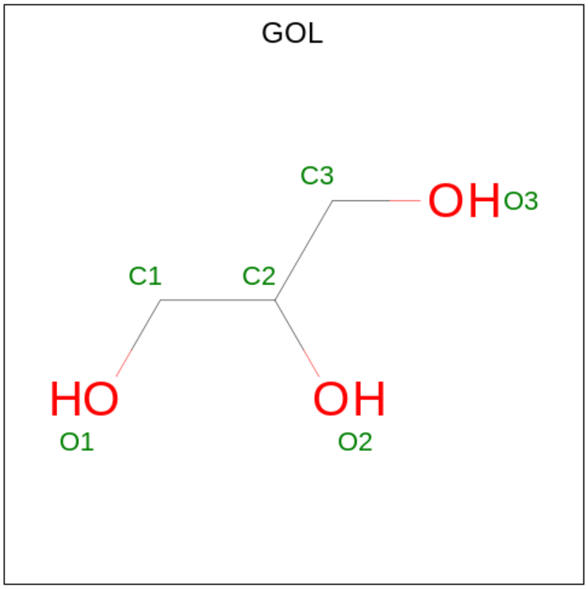

Mol	Chain	Residues	Atoms	ZeroOcc	AltConf
4	A	1	Total C O<br>6 3 3	0	0
4	B	1	Total C O<br>6 3 3	0	0

- Molecule 5 is water.

Mol	Chain	Residues	Atoms	ZeroOcc	AltConf
5	A	303	Total O<br>303 303	0	0
5	B	291	Total O<br>291 291	0	0

For Manuscript Review

3 Residue-property plots ⓘ

These plots are drawn for all protein, RNA, DNA and oligosaccharide chains in the entry. The first graphic for a chain summarises the proportions of the various outlier classes displayed in the second graphic. The second graphic shows the sequence view annotated by issues in geometry and electron density. Residues are color-coded according to the number of geometric quality criteria for which they contain at least one outlier: green = 0, yellow = 1, orange = 2 and red = 3 or more. A red dot above a residue indicates a poor fit to the electron density (RSRZ > 2). Stretches of 2 or more consecutive residues without any outlier are shown as a green connector. Residues present in the sample, but not in the model, are shown in grey.

• Molecule 1: Dihydroxy-acid dehydratase, chloroplastic

Chain A:

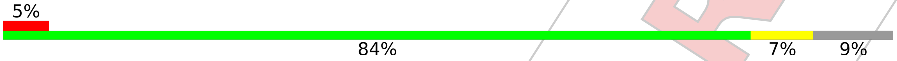

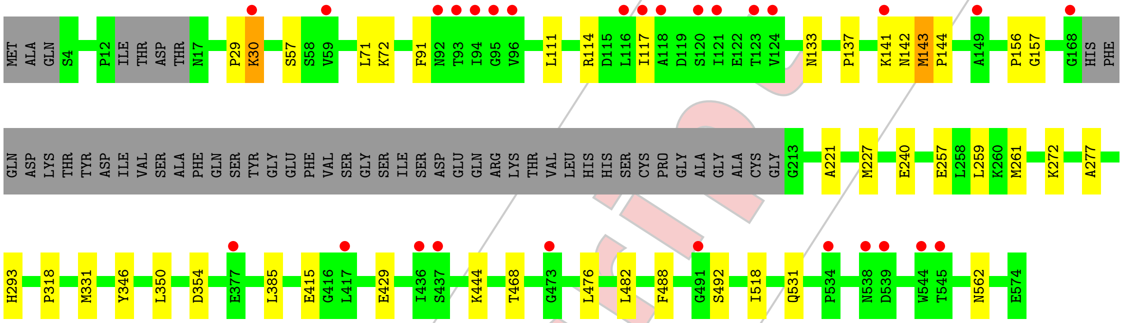

• Molecule 1: Dihydroxy-acid dehydratase, chloroplastic

Chain B:

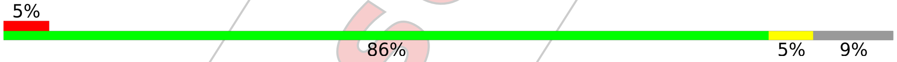

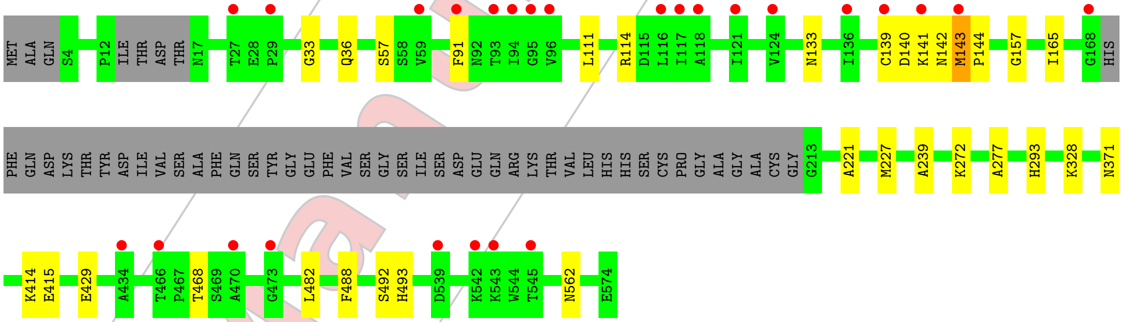

For Manuscript Review

4 Data and refinement statistics (i)

Property	Value	Source
Space group	P 41 21 2	Depositor
Cell constants<br>a, b, c, α, β, γ	135.12Å 135.12Å 136.44Å<br>90.00° 90.00° 90.00°	Depositor
Resolution (Å)	48.00 – 1.93<br>48.00 – 1.93	Depositor<br>EDS
% Data completeness<br>(in resolution range)	99.7 (48.00-1.93)<br>100.0 (48.00-1.93)	Depositor<br>EDS
R_{merge}	0.11	Depositor
R_{sym}	(Not available)	Depositor
$< I/\sigma(I) >$ [1]	1.87 (at 1.92Å)	Xtriage
Refinement program	PHENIX 1.20.1_4487	Depositor
R, R_{free}	0.182 , 0.208<br>0.180 , 0.207	Depositor<br>DCC
R_{free} test set	4718 reflections (4.97%)	wwPDB-VP
Wilson B-factor (Å^2)	28.8	Xtriage
Anisotropy	0.430	Xtriage
Bulk solvent k_{sol}(e/Å^3), B_{sol}(Å^2)	0.36 , 47.6	EDS
L-test for twinning[2]	$< \|L\| >$ = 0.49, $< L^2 >$ = 0.32	Xtriage
Estimated twinning fraction	0.000 for -h,l,k<br>0.000 for -l,-k,-h	Xtriage
F_o,F_c correlation	0.97	EDS
Total number of atoms	8582	wwPDB-VP
Average B, all atoms (Å^2)	33.0	wwPDB-VP

Xtriage's analysis on translational NCS is as follows: *The analyses of the Patterson function reveals a significant off-origin peak that is 83.20 % of the origin peak, indicating pseudo-translational symmetry. The chance of finding a peak of this or larger height randomly in a structure without pseudo-translational symmetry is equal to 2.3043e-07. The detected translational NCS is most likely also responsible for the elevated intensity ratio.*

[1] Intensities estimated from amplitudes.

[2] Theoretical values of $< |L| >$, $< L^2 >$ for acentric reflections are 0.5, 0.333 respectively for untwinned datasets, and 0.375, 0.2 for perfectly twinned datasets.

For Manuscript Review

5 Model quality (i)

5.1 Standard geometry (i)

Bond lengths and bond angles in the following residue types are not validated in this section: ACT, FES, GOL

The Z score for a bond length (or angle) is the number of standard deviations the observed value is removed from the expected value. A bond length (or angle) with $|Z| > 5$ is considered an outlier worth inspection. RMSZ is the root-mean-square of all Z scores of the bond lengths (or angles).

Mol	Chain	Bond lengths		Bond angles	
		RMSZ	#$\|Z\|$ >5	RMSZ	#$\|Z\|$ >5
1	A	0.43	1/4051 (0.0%)	0.67	5/5470 (0.1%)
1	B	0.40	0/4051	0.62	0/5470
All	All	0.42	1/8102 (0.0%)	0.64	5/10940 (0.0%)

Chiral center outliers are detected by calculating the chiral volume of a chiral center and verifying if the center is modelled as a planar moiety or with the opposite hand.A planarity outlier is detected by checking planarity of atoms in a peptide group, atoms in a mainchain group or atoms of a sidechain that are expected to be planar.

Mol	Chain	#Chirality outliers	#Planarity outliers
1	A	0	1

All (1) bond length outliers are listed below:

Mol	Chain	Res	Type	Atoms	Z	Observed(Å)	Ideal(Å)
1	A	30	LYS	CD-CE	-6.43	1.35	1.51

All (5) bond angle outliers are listed below:

Mol	Chain	Res	Type	Atoms	Z	Observed(^{o})	Ideal(^{o})
1	A	30	LYS	CA-CB-CG	8.01	131.02	113.40
1	A	29	PRO	C-N-CA	-7.46	103.04	121.70
1	A	30	LYS	N-CA-CB	7.39	123.90	110.60
1	A	30	LYS	CB-CG-CD	-6.12	95.68	111.60
1	A	30	LYS	CB-CA-C	-5.31	99.78	110.40

There are no chirality outliers.

All (1) planarity outliers are listed below:

For Manuscript Review

Mol	Chain	Res	Type	Group
1	A	30	LYS	Mainchain

5.2 Too-close contacts (i)

In the following table, the Non-H and H(model) columns list the number of non-hydrogen atoms and hydrogen atoms in the chain respectively. The H(added) column lists the number of hydrogen atoms added and optimized by MolProbity. The Clashes column lists the number of clashes within the asymmetric unit, whereas Symm-Clashes lists symmetry-related clashes.

Mol	Chain	Non-H	H(model)	H(added)	Clashes	Symm-Clashes
1	A	3980	0	4005	33	0
1	B	3980	0	4005	27	1
2	A	4	0	0	0	0
2	B	4	0	0	2	0
3	A	4	0	3	0	0
3	B	4	0	3	0	0
4	A	6	0	8	0	0
4	B	6	0	8	0	0
5	A	303	0	0	6	0
5	B	291	0	0	6	0
All	All	8582	0	8032	59	1

The all-atom clashscore is defined as the number of clashes found per 1000 atoms (including hydrogen atoms). The all-atom clashscore for this structure is 4.

All (59) close contacts within the same asymmetric unit are listed below, sorted by their clash magnitude.

Atom-1	Atom-2	Interatomic distance (Å)	Clash overlap (Å)
1:A:562:ASN:HD21	1:B:562:ASN:HD21	1.16	0.88
1:A:111:LEU:HA	1:A:141[B]:LYS:HG3	1.68	0.74
1:B:111:LEU:HA	1:B:141[B]:LYS:HG3	1.71	0.72
1:A:143[B]:MET:HG3	5:A:706:HOH:O	1.89	0.71
1:B:143[B]:MET:HG3	5:B:702:HOH:O	1.90	0.71
1:A:272:LYS:HD3	1:A:354:ASP:OD2	1.95	0.67
1:A:114:ARG:HH11	1:A:293:HIS:HD1	1.46	0.64
1:B:143[B]:MET:HE1	1:B:221:ALA:HA	1.80	0.62
1:B:114:ARG:HH11	1:B:293:HIS:HD1	1.50	0.59
1:A:429:GLU:OE2	5:A:701:HOH:O	2.17	0.58
1:A:143[B]:MET:HE1	1:A:221:ALA:HA	1.85	0.57
1:A:468:THR:HG21	1:A:492:SER:OG	2.04	0.56
1:B:142[B]:ASN:HB2	5:B:703:HOH:O	2.06	0.56

Continued on next page...

For Manuscript Review

Continued from previous page...

Atom-1	Atom-2	Interatomic distance (Å)	Clash overlap (Å)
1:B:143[B]:MET:HB2	1:B:144:PRO:HD3	1.91	0.52
1:A:142[B]:ASN:HB2	5:A:712:HOH:O	2.09	0.52
1:B:468:THR:HG21	1:B:492:SER:OG	2.10	0.50
1:A:227:MET:HB2	1:A:277:ALA:HB2	1.96	0.48
1:B:140[A]:ASP:HA	2:B:601:FES:S1	2.53	0.48
1:B:165:ILE:HD13	1:B:239:ALA:HB3	1.95	0.48
1:A:57:SER:HB3	1:A:91:PHE:CZ	2.49	0.48
1:A:143[B]:MET:HE1	1:A:221:ALA:CA	2.44	0.48
1:A:257:GLU:O	1:A:261:MET:HG3	2.14	0.48
1:A:518:ILE:HB	1:A:531:GLN:HB2	1.96	0.47
1:A:142[B]:ASN:OD1	1:A:142[B]:ASN:N	2.46	0.47
1:A:143[B]:MET:HB2	1:A:144:PRO:HD3	1.97	0.47
1:B:57:SER:HB3	1:B:91:PHE:CZ	2.49	0.47
1:B:429:GLU:OE2	5:B:701:HOH:O	2.20	0.47
1:B:139[A]:CYS:O	2:B:601:FES:S1	2.74	0.46
1:B:33:GLY:HA2	1:B:36:GLN:NE2	2.31	0.46
1:A:142[B]:ASN:O	1:A:143[B]:MET:HB2	2.16	0.45
1:A:482:LEU:HG	1:A:488:PHE:CZ	2.51	0.45
1:B:493:HIS:HD2	5:B:719:HOH:O	1.98	0.45
1:A:141[B]:LYS:HB2	5:A:858:HOH:O	2.17	0.45
1:A:117:ILE:HD12	1:A:142[A]:ASN:HB2	1.99	0.45
1:A:318:PRO:HG3	1:A:385:LEU:HD12	1.99	0.44
1:B:143[A]:MET:HG2	1:B:144:PRO:HD3	1.98	0.44
1:A:331:MET:HA	1:A:331:MET:HE2	1.99	0.44
1:A:156:PRO:HG2	1:A:259:LEU:HD23	2.00	0.44
1:B:141[B]:LYS:HA	1:B:141[B]:LYS:HD3	1.45	0.43
1:B:328:LYS:HB3	1:B:328:LYS:HE2	1.94	0.43
1:A:142[B]:ASN:OD1	5:A:702:HOH:O	2.21	0.43
1:B:140[A]:ASP:HB2	5:B:777:HOH:O	2.18	0.43
1:A:141[B]:LYS:O	1:A:144:PRO:HD2	2.19	0.43
1:B:142[B]:ASN:OD1	1:B:142[B]:ASN:N	2.51	0.43
1:A:133:ASN:O	1:A:157:GLY:HA2	2.19	0.43
1:A:137:PRO:HB2	1:A:143[B]:MET:HG2	2.01	0.42
1:B:141[B]:LYS:HB2	5:B:823:HOH:O	2.18	0.42
1:A:143[A]:MET:HG2	1:A:144:PRO:HD3	2.02	0.42
1:B:133:ASN:O	1:B:157:GLY:HA2	2.20	0.42
1:B:227:MET:HB2	1:B:277:ALA:HB2	2.01	0.42
1:B:482:LEU:HG	1:B:488:PHE:CE2	2.55	0.42
1:A:71:LEU:HB2	5:A:831:HOH:O	2.19	0.41
1:A:72:LYS:NZ	1:A:240:GLU:OE2	2.52	0.41
1:A:346:TYR:CE1	1:A:350:LEU:HD21	2.55	0.41

Continued on next page...

For Manuscript Review

Continued from previous page...

Atom-1	Atom-2	Interatomic distance (Å)	Clash overlap (Å)
1:A:444:LYS:HG3	1:A:476:LEU:HD21	2.02	0.41
1:B:142[B]:ASN:O	1:B:143[B]:MET:HB2	2.20	0.41
1:B:482:LEU:HG	1:B:488:PHE:CZ	2.55	0.41
1:A:482:LEU:HG	1:A:488:PHE:CE2	2.55	0.41
1:B:272:LYS:HE2	1:B:272:LYS:HB2	1.95	0.41

All (1) symmetry-related close contacts are listed below. The label for Atom-2 includes the symmetry operator and encoded unit-cell translations to be applied.

Atom-1	Atom-2	Interatomic distance (Å)	Clash overlap (Å)
1:B:371:ASN:OD1	1:B:414:LYS:NZ[4_544]	2.11	0.09

5.3 Torsion angles (i)

5.3.1 Protein backbone (i)

In the following table, the Percentiles column shows the percent Ramachandran outliers of the chain as a percentile score with respect to all X-ray entries followed by that with respect to entries of similar resolution.

The Analysed column shows the number of residues for which the backbone conformation was analysed, and the total number of residues.

Mol	Chain	Analysed	Favoured	Allowed	Outliers	Percentiles	
1	A	531/574 (92%)	512 (96%)	17 (3%)	2 (0%)	34	24
1	B	531/574 (92%)	511 (96%)	18 (3%)	2 (0%)	34	24
All	All	1062/1148 (92%)	1023 (96%)	35 (3%)	4 (0%)	47	24

All (4) Ramachandran outliers are listed below:

Mol	Chain	Res	Type
1	A	143[A]	MET
1	A	143[B]	MET
1	B	143[A]	MET
1	B	143[B]	MET

For Manuscript Review

5.3.2 Protein sidechains (i)

In the following table, the Percentiles column shows the percent sidechain outliers of the chain as a percentile score with respect to all X-ray entries followed by that with respect to entries of similar resolution.

The Analysed column shows the number of residues for which the sidechain conformation was analysed, and the total number of residues.

Mol	Chain	Analysed	Rotameric	Outliers	Percentiles	
1	A	437/466 (94%)	436 (100%)	1 (0%)	93	94
1	B	437/466 (94%)	436 (100%)	1 (0%)	93	94
All	All	874/932 (94%)	872 (100%)	2 (0%)	93	94

All (2) residues with a non-rotameric sidechain are listed below:

Mol	Chain	Res	Type
1	A	415	GLU
1	B	415	GLU

Sometimes sidechains can be flipped to improve hydrogen bonding and reduce clashes. All (4) such sidechains are listed below:

Mol	Chain	Res	Type
1	B	36	GLN
1	B	493	HIS
1	B	538	ASN
1	B	562	ASN

5.3.3 RNA (i)

There are no RNA molecules in this entry.

5.4 Non-standard residues in protein, DNA, RNA chains (i)

There are no non-standard protein/DNA/RNA residues in this entry.

5.5 Carbohydrates (i)

There are no monosaccharides in this entry.

For Manuscript Review

5.6 Ligand geometry (i)

6 ligands are modelled in this entry.

In the following table, the Counts columns list the number of bonds (or angles) for which Mogul statistics could be retrieved, the number of bonds (or angles) that are observed in the model and the number of bonds (or angles) that are defined in the Chemical Component Dictionary. The Link column lists molecule types, if any, to which the group is linked. The Z score for a bond length (or angle) is the number of standard deviations the observed value is removed from the expected value. A bond length (or angle) with $|Z| > 2$ is considered an outlier worth inspection. RMSZ is the root-mean-square of all Z scores of the bond lengths (or angles).

Mol	Type	Chain	Res	Link	Bond lengths			Bond angles		
					Counts	RMSZ	$\#\|Z\| > 2$	Counts	RMSZ	$\#\|Z\| > 2$
3	ACT	A	602	-	3,3,3	1.28	0	3,3,3	1.38	0
4	GOL	A	603	-	5,5,5	0.24	0	5,5,5	0.68	0
2	FES	B	601	1	0,4,4	-	-	-		
2	FES	A	601	1	0,4,4	-	-	-		
4	GOL	B	603	-	5,5,5	0.20	0	5,5,5	0.66	0
3	ACT	B	602	-	3,3,3	1.61	1 (33%)	3,3,3	1.57	1 (33%)

In the following table, the Chirals column lists the number of chiral outliers, the number of chiral centers analysed, the number of these observed in the model and the number defined in the Chemical Component Dictionary. Similar counts are reported in the Torsion and Rings columns. '-' means no outliers of that kind were identified.

Mol	Type	Chain	Res	Link	Chirals	Torsions	Rings
2	FES	B	601	1	-	-	0/1/1/1
4	GOL	A	603	-	-	2/4/4/4	-
2	FES	A	601	1	-	-	0/1/1/1
4	GOL	B	603	-	-	2/4/4/4	-

All (1) bond length outliers are listed below:

Mol	Chain	Res	Type	Atoms	Z	Observed(Å)	Ideal(Å)
3	B	602	ACT	CH3-C	2.51	1.59	1.49

All (1) bond angle outliers are listed below:

Mol	Chain	Res	Type	Atoms	Z	Observed(^{o})	Ideal(^{o})
3	B	602	ACT	O-C-CH3	-2.02	114.48	122.33

There are no chirality outliers.

All (4) torsion outliers are listed below:

Mol	Chain	Res	Type	Atoms
4	A	603	GOL	O1-C1-C2-O2
4	A	603	GOL	O1-C1-C2-C3
4	B	603	GOL	O1-C1-C2-O2
4	B	603	GOL	O1-C1-C2-C3

There are no ring outliers.

1 monomer is involved in 2 short contacts:

Mol	Chain	Res	Type	Clashes	Symm-Clashes
2	B	601	FES	2	0

The following is a two-dimensional graphical depiction of Mogul quality analysis of bond lengths, bond angles, torsion angles, and ring geometry for all instances of the Ligand of Interest. In addition, ligands with molecular weight > 250 and outliers as shown on the validation Tables will also be included. For torsion angles, if less then 5% of the Mogul distribution of torsion angles is within 10 degrees of the torsion angle in question, then that torsion angle is considered an outlier. Any bond that is central to one or more torsion angles identified as an outlier by Mogul will be highlighted in the graph. For rings, the root-mean-square deviation (RMSD) between the ring in question and similar rings identified by Mogul is calculated over all ring torsion angles. If the average RMSD is greater than 60 degrees and the minimal RMSD between the ring in question and any Mogul-identified rings is also greater than 60 degrees, then that ring is considered an outlier. The outliers are highlighted in purple. The color gray indicates Mogul did not find sufficient equivalents in the CSD to analyse the geometry.

For Manuscript Review

Ligand FES B 601

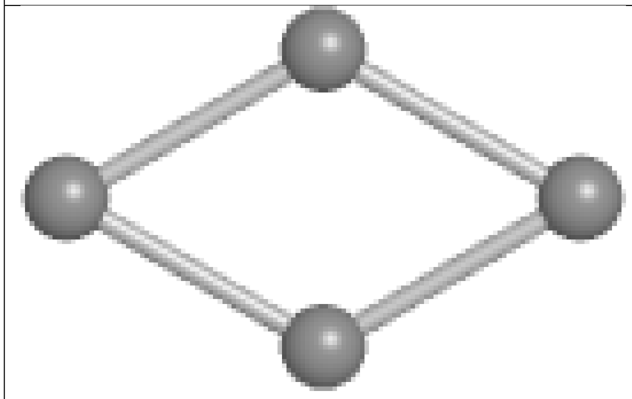

Bond lengths

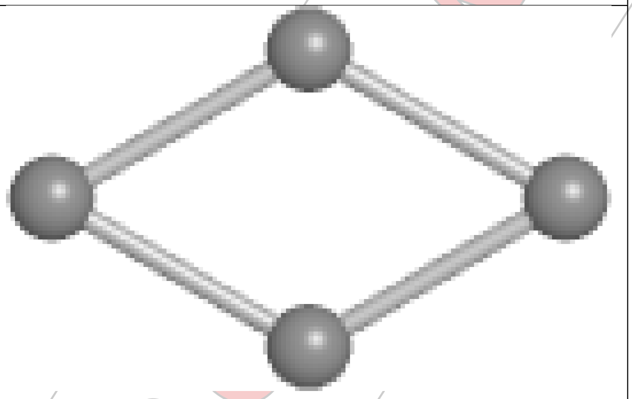

Bond angles

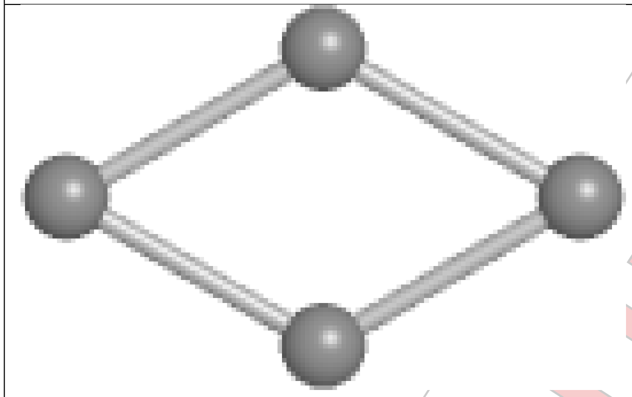

Torsions

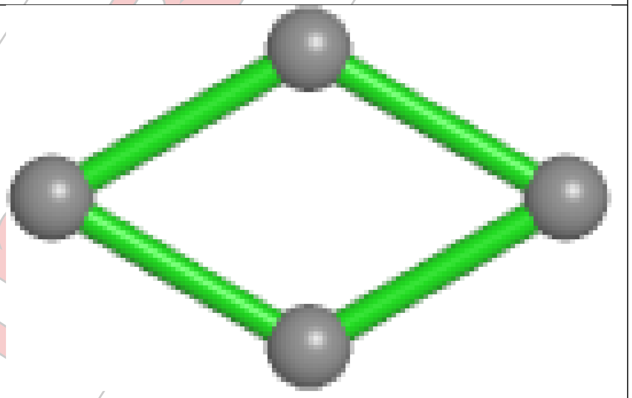

Rings

For Manuscript Review

Ligand FES A 601

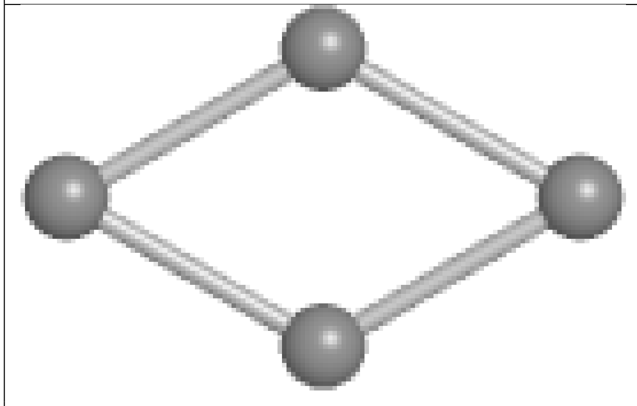

Bond lengths

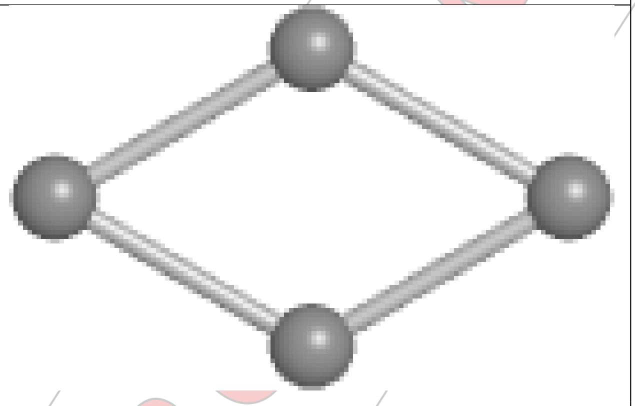

Bond angles

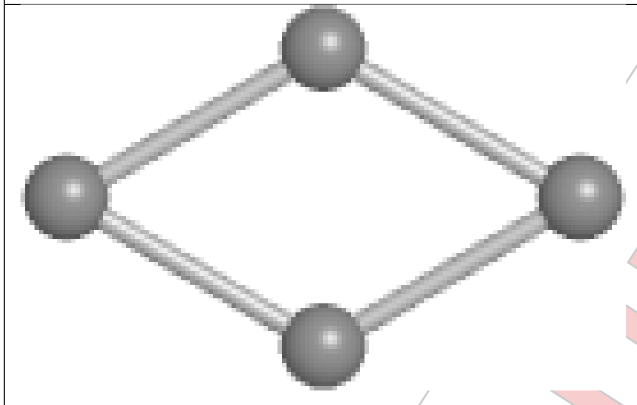

Torsions

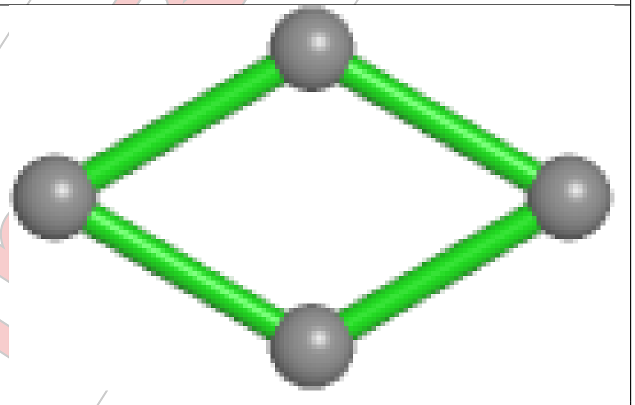

Rings

5.7 Other polymers (i)

There are no such residues in this entry.

5.8 Polymer linkage issues (i)

There are no chain breaks in this entry.

For Manuscript Review

6 Fit of model and data (i)

6.1 Protein, DNA and RNA chains (i)

In the following table, the column labelled '#RSRZ> 2' contains the number (and percentage) of RSRZ outliers, followed by percent RSRZ outliers for the chain as percentile scores relative to all X-ray entries and entries of similar resolution. The OWAB column contains the minimum, median, 95th percentile and maximum values of the occupancy-weighted average B-factor per residue. The column labelled 'Q< 0.9' lists the number of (and percentage) of residues with an average occupancy less than 0.9.

Mol	Chain	Analysed	<RSRZ>	#RSRZ>2			OWAB(Å^2)	Q<0.9
1	A	523/574 (91%)	0.23	28 (5%)	25	32	19, 30, 49, 85	0
1	B	523/574 (91%)	0.25	26 (4%)	28	36	20, 31, 51, 87	0
All	All	1046/1148 (91%)	0.24	54 (5%)	27	34	19, 30, 50, 87	0

All (54) RSRZ outliers are listed below:

Mol	Chain	Res	Type	RSRZ
1	B	168	GLY	5.7
1	A	168	GLY	5.3
1	B	93[A]	THR	4.3
1	A	141[A]	LYS	3.9
1	A	117	ILE	3.8
1	A	30	LYS	3.8
1	B	141[A]	LYS	3.6
1	A	94	ILE	3.6
1	B	118	ALA	3.4
1	B	117	ILE	3.2
1	A	121	ILE	3.1
1	A	59	VAL	3.0
1	B	121	ILE	3.0
1	A	120[A]	SER	3.0
1	B	545	THR	3.0
1	A	491	GLY	2.9
1	A	93[A]	THR	2.9
1	A	116	LEU	2.9
1	B	94	ILE	2.9
1	A	124	VAL	2.7
1	B	124	VAL	2.7
1	B	116	LEU	2.7
1	B	59	VAL	2.6
1	A	96	VAL	2.6

Continued on next page...

For Manuscript Review

Continued from previous page...

Mol	Chain	Res	Type	RSRZ
1	B	91	PHE	2.6
1	B	139[A]	CYS	2.6
1	B	539	ASP	2.6
1	B	96	VAL	2.4
1	A	95	GLY	2.4
1	B	473	GLY	2.4
1	A	473	GLY	2.3
1	A	545	THR	2.3
1	A	539	ASP	2.3
1	A	92	ASN	2.3
1	A	436	ILE	2.3
1	B	29	PRO	2.3
1	B	27	THR	2.2
1	A	437	SER	2.2
1	B	136	ILE	2.2
1	A	534	PRO	2.2
1	B	466	THR	2.2
1	A	377	GLU	2.2
1	A	544	TRP	2.2
1	B	143[A]	MET	2.2
1	B	95	GLY	2.2
1	A	118	ALA	2.1
1	B	543	LYS	2.1
1	B	434	ALA	2.1
1	B	470	ALA	2.1
1	A	538	ASN	2.1
1	A	123	THR	2.1
1	A	417	LEU	2.1
1	B	542	LYS	2.1
1	A	149	ALA	2.0

6.2 Non-standard residues in protein, DNA, RNA chains (i)

There are no non-standard protein/DNA/RNA residues in this entry.

6.3 Carbohydrates (i)

There are no monosaccharides in this entry.

For Manuscript Review

6.4 Ligands (i)

LIGAND-RSR INFOmissingINFO

6.5 Other polymers (i)

There are no such residues in this entry.

For Manuscript Review
