# Supplementary material for: Structural Bases of Dihydroxy Acid Dehydratase Inhibition and Biodesign for Self-Resistance
Source: Biodes Res. 2024 Nov 1;6:0046. doi: 10.34133/bdr.0046 (PMC11528067; doi:10.34133/bdr.0046)
Supplement: Supplementary 1 — Tables S1 to S7 Figs. S1 to S26 [file bdr.0046.f1.zip › SI revised-clean copydocx.docx]

**Supplementary material**

**Structural bases of dihydroxy acid dehydratase inhibition by aspterric acid and the mechanism of self-resistance by AstD**

Xin Zang^1,#^, Undramaa Bat-Erdene^2#^, Weixue Huang,^3^ Zhongshou Wu,^4,5^ Steve E. Jacobsen,^4-7^ Yi Tang^2,^*, and Jiahai Zhou^1^*

^1^CAS Key Laboratory of Quantitative Engineering Biology, Shenzhen Institute of Synthetic Biology, Shenzhen Institute of Advanced Technology, Chinese Academy of Sciences, Shenzhen 518055, China

^2^Department of Chemical and Biomolecular Engineering, University of California Los Angeles, Los Angeles, CA, USA

^3^State Key Laboratory of Chemical Biology, Shanghai Institute of Organic Chemistry, Chinese Academy of Sciences, Shanghai 200032, China

^4^Department of Molecular Cell and Developmental Biology, University of California, Los Angeles, California 90095, United States

^5^Howard Hughes Medical Institute, University of California, Los Angeles, California 90095, United States

^6^Eli & Edythe Broad Center of Regenerative Medicine & Stem Cell Research, University of California, Los Angeles, California 90095, United States

^7^Department of Biological Chemistry, University of California, Los Angeles, California 90095, United States

^#^ these authors contributed equally to this work

* corresponding authors

Email: [yitang@ucla.edu](mailto:yitang@ucla.edu); [jiahai@siat.ac.cn](mailto:jiahai@siat.ac.cn)

**Table of Contents**

1. **Supplementary Tables** ......................................................................................................... 3

**Table S1**. Primers and plasmids used in this study ..................................................................... 3

**Table S2**. X-ray data collection and refinement statistics of *Ath*DHAD-AA complex ................... 4

**Table S3**. X-ray data collection and refinement statistics of AstD ............................................... 5

**Table S4**. X-ray data collection and refinement statistics of *Ath*DHAD-V496W and *Ath*DHAD-V497F .......................................................................................................................... 6

**Table S5**. X-ray data collection and refinement statistics of *Ath*DHAD-I177F and *Ath*DHAD-V178W ......................................................................................................................... 7

**Table S6**. Summary of *Ath*DHAD mutations ................................................................................ 8

**Table S7**. Summary of *k*_cat_ and *K*_M_ of *Ath*DHAD and its mutants ................................................. 9

1. **Supplementary Figures** ..................................................................................................... 10

**Figure S1**. The structure of *Ath*DHAD-AA complex and simulated annealing Fo-Fc maps of AA and 2Fe-2S............................................................................................................... 10

**Figure S2.** The alignment of *Ath*DHAD (blue) and *Ath*DHAD-AA complex (purple) around the substrate channel. ..................................................................................................... 11

**Figure S3**. Composition of the entry channel of *Ath*DHAD ........................................................ 12

**Figure S4.** Surface representation of the narrowest part of the substrate channel of *Ath*DHAD, AthDHAD-AA and AstD ............................................................................................ 13

**Figure S5**. The overall alignment of *Ath*DHAD with its four mutants .......................................... 14

**Figure S6**. Shared enzymes within the branched chain amino acid biosynthetic pathway ........ 15

**Figure S7**. Biochemical assay scheme for dihydroxy acid dehydratase activity ......................... 16

**Figure S8**. SDS-PAGE of purified *Ath*DHAD and its mutants ..................................................... 17

**Figure S9**. *Ath*DHAD biochemical assay results ........................................................................ 18

**Figure S10**. *Ath*DHAD-I177L mutant biochemical assay results ................................................ 19

**Figure S11**. *Ath*DHAD-I177F mutant biochemical assay results ............................................... 20

**Figure S12**. *Ath*DHAD-V178L mutant biochemical assay results ............................................... 21

**Figure S13**. *Ath*DHAD-V178I mutant biochemical assay results ................................................ 22

**Figure S14**. *Ath*DHAD-V178F mutant biochemical assay results ............................................... 23

**Figure S15**. *Ath*DHAD-V178W mutant biochemical assay results ............................................. 24

**Figure S16**. *Ath*DHAD-V496L mutant biochemical assay results .............................................. 25

**Figure S17**. *Ath*DHAD-V496I mutant biochemical assay results ............................................... 26

**Figure S18**. *Ath*DHAD-V496F mutant biochemical assay results .............................................. 27

**Figure S19**. *Ath*DHAD-V497L mutant biochemical assay results .............................................. 28

**Figure S20**. *Ath*DHAD-V497I mutant biochemical assay results ............................................... 29

**Figure S21**. *Ath*DHAD-V497F mutant biochemical assay results .............................................. 30

**Figure S22**. *Ath*DHAD-I177L-V496L mutant biochemical assay results .................................... 31

**Figure S23**. *Ath*DHAD-I177L-V497L mutant biochemical assay results .................................... 32

**Figure S24**. *Ath*DHAD-V178L-V497L mutant biochemical assay results .................................. 33

**Figure S25**. 2-keto-isovalerate (KIV) calibration curve used for calculations of DHAD product concentration ......................................................................................................... 34

**Figure S26** Aspterric acid effect on *A. thaliana* carrying V178L or V496L mutant *Ath*DHAD .... 35

**3. Supplemental References** …………………………………………………………………........ 36

**1. Supplementary tables**

**Table S1**. Primers and plasmids used in this study

| Plasmid name | Primers (forward/reverse) | Mutation introduced |
| --- | --- | --- |
| pET28a | n/a | n/a |
| pEUB10001 | atggctagcgcccaatccgtaaccgctg | *Ath*DHAD cloning |
|  | atagcggccgcttactcgtcagtcacacatccatctg |  |
| pEUB10002 | gacatatgaccttgtatctgctttccagag | I177L |
|  | cagatacaaggtcatatgtcttgtcttgaaaatg |  |
| pEUB10003 | caagacatatgactttgtatctgctttccagagttatggag | I177F |
|  | ggaaagcagatacccagtcatatgtcttgtcttgaaaatgg |  |
| pEUB10004 | gacaagacatatgactgggtatctgctttccagagttatggag | I177W |
|  | ggaaagcagatacccagtcatatgtcttgtcttgaaaatgg |  |
| pEUB10005 | gacatatgacattctgtctgctttccagagttatggagaatttg | V178L |
|  | ctggaaagcagacagaatgtcatatgtcttgtcttgaaaatg |  |
| pEUB10006 | gacatatgacattatttctgctttccagagttatggagaatttg | V178I |
|  | ctggaaagcagaaataatgtcatatgtcttgtcttgaaaatg |  |
| pEUB10007 | gacatatgacattttttctgctttccagagttatggagaatttg | V178F |
|  | ctggaaagcagaaaaaatgtcatatgtcttgtcttgaaaatg |  |
| pEUB10008 | gacatatgacatttggtctgctttccagagttatggagaatttg | V178W |
|  | ctggaaagcagaccaaatgtcatatgtcttgtcttgaaaatg |  |
| pEUB10009 | cacggttttcttgttggccacatttgc | V496L |
|  | gccaacaagaaaaccgtgtgacccaccag |  |
| pEUB10010 | cacggttttattgttggccacatttgc | V496I |
|  | gccaacaataaaaccgtgtgacccaccag |  |
| pEUB10011 | cacggtttttttgttggccacatttgccc | V496F |
|  | gccaacaaaaaaaccgtgtgacccacc |  |
| pEUB10012 | cacacggtttttgggttggccacatttgccctgaag | V496W |
|  | gtggccaacccaaaaaccgtgtgacccaccag |  |
| pEUB10013 | cacggttttgttcttggccacatttgccc | V497L |
|  | gtggccaagaacaaaaccgtgtgacc |  |
| pEUB10014 | cacggttttgttattggccacatttgccc | V497I |
|  | gtggccaataacaaaaccgtgtgaccc |  |
| pEUB10015 | cacggttttgtttttggccacatttgccc | V497F |
|  | gtggccaaaaacaaaaccgtgtgaccc |  |
| pEUB10016 | cacacggttttgtttggggccacatttgccctgaag | V497W |
|  | caaatgtggccccaaacaaaaccgtgtgacccaccag |  |
| pEUB10017 | gacatatgaccttgtatctgctttccagag | I177L |
|  | cagatacaaggtcatatgtcttgtcttgaaaatg |  |
|  | cacggttttcttgttggccacatttgc | V496L |
|  | gccaacaagaaaaccgtgtgacccaccag |  |
| pEUB10018 | gacatatgaccttgtatctgctttccagag | I177L |
|  | cagatacaaggtcatatgtcttgtcttgaaaatg |  |
|  | cacggttttgttcttggccacatttgccc | V497L |
|  | gtggccaagaacaaaaccgtgtgacc |  |
| pEUB10018 | gacatatgacattctgtctgctttccagagttatggagaatttg | V178L |
|  | ctggaaagcagacagaatgtcatatgtcttgtcttgaaaatg |  |
|  | cacggttttgttcttggccacatttgccc | V497L |
|  | gtggccaagaacaaaaccgtgtgacc |  |

**Table S2.** X-ray data collection and refinement statistics of *Ath*DHAD-AA complex.

|  | *Ath*DHAD-AA |
| --- | --- |
| **Data collection** |  |
| Space group | *P4_2_2_1_2* |
| Cell dimensions |  |
| *a*, *b*, *c* (Å) | 135.8, 135.8, 66.64 |
| α, β, γ (°) | 90.00, 90.00, 90.00 |
| Resolution (Å) | 47.58-2.00 (2.07-2.00) * |
| *R*_sym_ or *R*_merge_ | 0.145 (1.007) |
| *I* / σ*I* | 8.62 (2) |
| Completeness (%) | 100.0 (100.0) |
| Redundancy | 7.5 (7.2) |
|  |  |
| **Refinement** |  |
| Resolution (Å) | 47.57-2.00 (2.07-2.00) |
| No. reflections | 42627 (4175) |
| *R*_work_ / *R*_free_ | 0.1809/0.2135 |
| No. atoms | 4566 |
| Protein | 4250 |
| Ligand/ion | 48 |
| Water | 268 |
| *B*-factors | 35.53 |
| Protein | 35.24 |
| Ligand/ion | 47.63 |
| Water | 37.96 |
| R.m.s. deviations |  |
| Bond lengths (Å) | 0.008 |
| Bond angles (°) | 0.93 |

*Number of xtals for each structure should be noted in footnote. *Values in parentheses are for highest-resolution shell.

[AU: Equations defining various *R*-values are standard and hence are no longer defined in the footnotes.]

[AU: Ramachandran statistics should be in Methods section at the end of Refinement subsection.]

[AU: Wavelength of data collection, temperature and beamline should all be in Methods section.]

**Table S3**. X-ray data collection and refinement statistics of AstD

|  | AstD |
| --- | --- |
| **Data collection** |  |
| Space group | *P2_1_2_1_2_1_* |
| Cell dimensions |  |
| *a*, *b*, *c* (Å) | 56.61, 66.27, 268.9 |
| α, β, γ (°) | 90.00, 90.00, 90.00 |
| Resolution (Å) | 50-2.30 (2.34-2.30) * |
| *R*_sym_ or *R*_merge_ | 0.185 (1.040) |
| *I* / σ*I* | 10.9 (1.9) |
| Completeness (%) | 99.9 (100.0) |
| Redundancy | 7.3 (6.8) |
|  |  |
| **Refinement** |  |
| Resolution (Å) | 44.83 - 2.28（2.37-2.28） |
| No. reflections | 45340 (3597) |
| *R*_work_ / *R*_free_ | 0.2007/0.2317 |
| No. atoms | 7293 |
| Protein | 6922 |
| Ligand/ion | 0 |
| Water | 371 |
| *B*-factors | 38.31 |
| Protein | 38.19 |
| Ligand/ion |  |
| Water | 40.47 |
| R.m.s. deviations |  |
| Bond lengths (Å) | 0.002 |
| Bond angles (°) | 0.64 |

*Number of xtals for each structure should be noted in footnote. *Values in parentheses are for highest-resolution shell.

[AU: Equations defining various *R*-values are standard and hence are no longer defined in the footnotes.]

[AU: Ramachandran statistics should be in Methods section at the end of Refinement subsection.]

[AU: Wavelength of data collection, temperature and beamline should all be in Methods section.]

**Table S4**. X-ray data collection and refinement statistics of *Ath*DHAD-V496W and *Ath*DHAD-V497F.

|  | *Ath*DHAD-V496W | *Ath*DHAD-V497F |
| --- | --- | --- |
| **Data collection** |  |  |
| Space group | *P4_2_2_1_2* | *P4_1_2_1_2* |
| Cell dimensions |  |  |
| *a*, *b*, *c* (Å) | 135.6, 135.6, 66.74 | 135.1, 135.1, 136.4 |
| α, β, γ (°) | 90.00, 90.00, 90.00 | 90.00, 90.00, 90.00 |
| Resolution (Å) | 47.53-1.75 (1.78-1.75) * | 48.00-1.93 (1.96-1.93) |
| *R*_sym_ or *R*_merge_ | 0.068 (0.788) | 0.108 (1.473) |
| *I* / σ*I* | 14.3(1.9) | 13.6 (2.0) |
| Completeness (%) | 98.9 (99.8) | 100.0 (99.9) |
| Redundancy | 6.6 (6.4) | 13.2 (13.4) |
|  |  |  |
| **Refinement** |  |  |
| Resolution (Å) | 37.60-1.75 (1.81-1.75) | 48.00-1.87 (1.99-1.93) |
| No. reflections | 62159 (6182) | 94685 (9326) |
| *R*_work_ / *R*_free_ | 0.1785/0.2113 | 0.1816/0.2078 |
| No. atoms | 4671 | 8582 |
| Protein | 4268 | 7960 |
| Ligand/ion | 16 | 28 |
| Water | 387 | 594 |
| *B*-factors | 19.66 | 33.36 |
| Protein | 18.40 | 32.84 |
| Ligand/ion | 21.36 | 44.27 |
| Water | 30.59 | 39.86 |
| R.m.s. deviations |  |  |
| Bond lengths (Å) | 0.007 | 0.008 |
| Bond angles (°) | 1.02 | 1.04 |

*Number of xtals for each structure should be noted in footnote. *Values in parentheses are for highest-resolution shell.

[AU: Equations defining various *R*-values are standard and hence are no longer defined in the footnotes.]

[AU: Ramachandran statistics should be in Methods section at the end of Refinement subsection.]

[AU: Wavelength of data collection, temperature and beamline should all be in Methods section.]

**Table S5**. X-ray data collection and refinement statistics of *Ath*DHAD-I177F and *Ath*DHAD-V178W.

|  | *Ath*DHAD-I177F | *Ath*DHAD-V178W |
| --- | --- | --- |
| **Data collection** |  |  |
| Space group | *P4_2_2_1_2* | *P4_2_2_1_2* |
| Cell dimensions |  |  |
| *a*, *b*, *c* (Å) | 135.5, 135.5, 66.18 | 135.9 135.9 66.38 |
| α, β, γ (°) | 90.00, 90.00, 90.00 | 90.00, 90.00, 90.00 |
| Resolution (Å) | 47.59-2.45 (2.55-2.45) * | 45.31-1.42 (1.44-1.42) |
| *R*_sym_ or *R*_merge_ | 0.104 (0.792) | 0.075 (1.341) |
| *I* / σ*I* | 9.4 (1.9) | 18.0 (2.0) |
| Completeness (%) | 96.7 (98.8) | 100.0 (100.0) |
| Redundancy | 4.8 (5.0) | 12.7 (10.0) |
|  |  |  |
| **Refinement** |  |  |
| Resolution (Å) | 38.94-2.45 (2.53- 2.45) | 42.98-1.42 (1.47-1.42) |
| No. reflections | 22491 (2265) | 116775 (11530) |
| *R*_work_ / *R*_free_ | 0.1967/0.2327 | 0.1811/ 0.1967 |
| No. atoms | 4163 | 4726 |
| Protein | 4083 | 4286 |
| Ligand/ion | 4 | 11 |
| Water | 18 | 429 |
| *B*-factors | 54.66 | 27.41 |
| Protein | 54.68 | 26.50 |
| Ligand/ion | 83.49 | 30.17 |
| Water | 43.6 | 36.41 |
| R.m.s. deviations |  |  |
| Bond lengths (Å) | 0.002 | 0.006 |
| Bond angles (°) | 0.55 | 1.10 |

*Number of xtals for each structure should be noted in footnote. *Values in parentheses are for highest-resolution shell.

[AU: Equations defining various *R*-values are standard and hence are no longer defined in the footnotes.]

[AU: Ramachandran statistics should be in Methods section at the end of Refinement subsection.]

[AU: Wavelength of data collection, temperature and beamline should all be in Methods section.]

^a^ Numbers in parentheses are values for the highest-resolution shell.

^b^*R*_merge_＝Σ*_hkl_*Σ_i_|*I*_i_ -〈*I*〉|/Σ*_hkl_*Σ_i_|〈*I*〉|, where I_i_ is the intensity for the *i*th measurement of an equivalent reflectionwith indices h, k, and l.

^c^*R*_free_ was calculated with the 5% of reflections set aside randomly throughout the refinement.

**Table S6**. Summary of *Ath*DHAD mutations

| Name of mutant enzymes | Mutation positions | Mutation |
| --- | --- | --- |
| I177L | Isoleucine  177 | Leu |
| I177F |  | Phe |
| I177W |  | Trp |
| V178L | Valine  178 | Leu |
| V178I |  | Ile |
| V178F |  | Phe |
| V178W |  | Trp |
| V496L | Valine  496 | Leu |
| V496I |  | Ile |
| V496F |  | Phe |
| V496L |  | Trp |
| V497L | Valine  497 | Leu |
| V497I |  | Ile |
| V497F |  | Phe |
| V497W |  | Trp |
| I177/V496L | Isoleucine 177  Valine 496 | Leu |
| I177/V497L | Isoleucine 177  Valine 497 | Leu |
| V178/V497L | Valine 178  Valine 497 | Leu |

**Table S7**. Summary of *k*_cat_ and *K*_M_ of *Ath*DHAD and its mutants

N/A : the biochemical data was not obtainable

**2. Supplementary figures**

**Figure S1**. The structure of *Ath*DHAD-AA complex

(a). Two domains of *Ath*DHAD. The N domain (1-388) is in blue and the C domain (394-end) is in green.

(b). The coordinating residues around Mg^2+^ (magentas) and [2Fe-2S].

(c). Simulated annealing Fo-Fc map of 2Fe-2S in the *Ath*DHAD-AA complex crystal structure, contoured at 5.0 σ.

(d). Simulated annealing Fo-Fc map of AA in the *Ath*DHAD-AA complex crystal structure, contoured at 1.5 σ.

**Figure S2.** The alignment of *Ath*DHAD (blue) and *Ath*DHAD-AA complex (purple) around the substrate channel.

(a). The binding of AA made the α-helix (178-200) of *Ath*DHAD-AA complex structure shift away from G490 about 0.6 Å.

(b). The alignment of the coordination changes around Mg^2+^between *Ath*DHAD (violet and *Ath*DHAD-AA (blue).

(c). The alignment of the sticks at the active chamber between *Ath*DHAD (violet) and *Ath*DHAD-AA (blue).


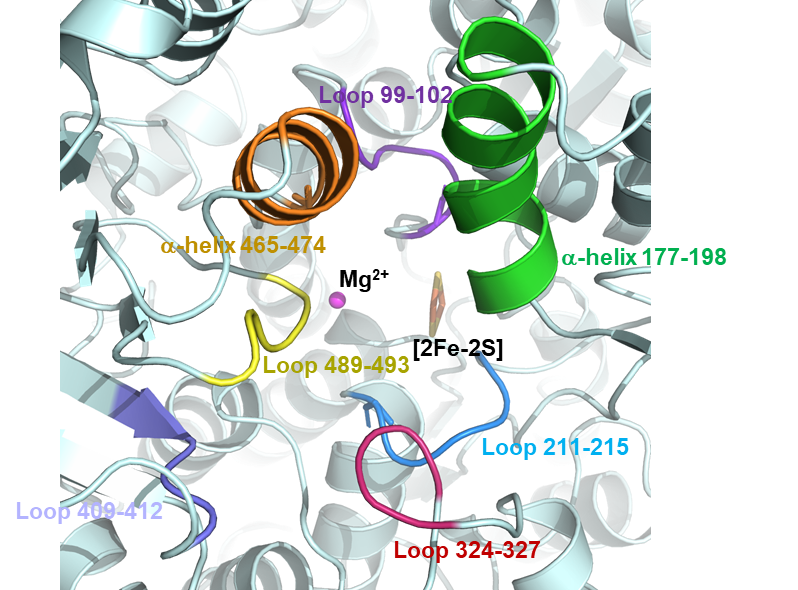


**Figure S3**. Composition of the entry channel of *Ath*DHAD. The entry channel consists of 6 parts, including α-helices 177-198 (in green), loop 211-215 (in blue), loop 324-327 (in red), loop 409-412 (in lavender), loop 489-493 (in yellow), α-helices 465-474 (in orange), loop 99-102 (in purple).

**Figure S4.** Surface representation of the narrowest part of the substrate channel of AthDHAD-AA (a), *Ath*DHAD (b) and AstD (c). These picture were made by Chimera^10^.

(a). The narrowest site of the substrate channel of *Ath*DHAD-AA complex is consist of G490 and V178. The corresponding diatance is 8.6 Å.

(b). The narrowest site of the substrate channel of *Ath*DHAD is consist of G490 and V178. The distance between these two residues is 8.0 Å.

(c). The narrowest site of the substrate channel of AstD is consist of G512 and V200. The diatance between these two residues is 7.7 Å.

**Figure S5**. The overall alignment of *Ath*DHAD with its four mutants.

(a) The overall structure alignment of *Ath*DHAD (blue) with *Ath*DHAD-V496W (darkgreen). The RMSD value is 0.143 Å for 1032 core C_α_ atoms.

(b) The overall structure alignment of *Ath*DHAD (blue) with *Ath*DHAD-V497F (yellow). The RMSD value is 0.276 Å for 982 core C_α_ atoms.

(c) The overall structure alignment of *Ath*DHAD (blue) with *Ath*DHAD-I177F (orange). The RMSD value is 0.218 Å for 944 core C_α_ atoms.

(d) The overall structure alignment of *Ath*DHAD (blue) with *Ath*DHAD-V178W (bright purple). The RMSD value is 0.130 Å for 976 core C_α_ atoms

**Figure S6**. Shared enzymes within the branched chain amino acid biosynthetic pathway

**Figure S7**. Biochemical assay scheme for dihydroxy acid dehydratase activity

**Figure S8**. SDS-PAGE of purified *Ath*DHAD and its mutants

**Figure S9**. *Ath*DHAD biochemical assay results. **A**. Reaction rate across different concentrations of α,β-dihydroxyisovalerate (DHI). **B**. Reaction rate across different concentrations of α,β-dihydroxymethylvalerate (DHMV). **C**. Inhibition percentage of 0.5 μM *Ath*DHAD as a function of aspterric acid (AA) concentration.

**Figure S10**. *Ath*DHAD-I177L mutant biochemical assay results. **A**. Reaction rate across different concentrations of α,β-dihydroxyisovalerate (DHI). **B**. Reaction rate across different concentrations of α,β-dihydroxymethylvalerate (DHMV). **C**. Inhibition percentage of 0.5 μM *Ath*DHAD-I177L as a function of aspterric acid (AA) concentration.

**Figure S11**. *Ath*DHAD-I177F mutant biochemical assay results. **A**. Reaction rate across different concentrations of α,β-dihydroxyisovalerate (DHI). **B**. Reaction rate across different concentrations of α,β-dihydroxymethylvalerate (DHMV). **C**. Inhibition percentage of 0.5 μM *Ath*DHAD-I177F as a function of aspterric acid (AA) concentration.

**Figure S12**. *Ath*DHAD-V178L mutant biochemical assay results. **A**. Reaction rate across different concentrations of α,β-dihydroxyisovalerate (DHI). **B**. Reaction rate across different concentrations of α,β-dihydroxymethylvalerate (DHMV). **C**. Inhibition percentage of 0.5 μM *Ath*DHAD-V178L as a function of aspterric acid (AA) concentration.

**Figure S13**. *Ath*DHAD-V178I mutant biochemical assay results. **A**. Reaction rate across different concentrations of α,β-dihydroxyisovalerate (DHI). **B**. Reaction rate across different concentrations of α,β-dihydroxymethylvalerate (DHMV). **C**. Inhibition percentage of 0.5 μM *Ath*DHAD-V178I as a function of aspterric acid (AA) concentration.

**Figure S14**. *Ath*DHAD-V178F mutant biochemical assay results. **A**. Reaction rate across different concentrations of α,β-dihydroxyisovalerate (DHI). **B**. Reaction rate across different concentrations of α,β-dihydroxymethylvalerate (DHMV). **C**. Inhibition percentage of 0.5 μM *Ath*DHAD-V178F as a function of aspterric acid (AA) concentration.

**Figure S15**. *Ath*DHAD-V178W mutant biochemical assay results. **A**. Reaction rate across different concentrations of α,β-dihydroxyisovalerate (DHI). **B**. Reaction rate across different concentrations of α,β-dihydroxymethylvalerate (DHMV). **C**. Inhibition percentage of 0.5 μM *Ath*DHAD-V178W as a function of aspterric acid (AA) concentration.

**Figure S16**. *Ath*DHAD-V496L mutant biochemical assay results. **A**. Reaction rate across different concentrations of α,β-dihydroxyisovalerate (DHI). **B**. Reaction rate across different concentrations of α,β-dihydroxymethylvalerate (DHMV). **C**. Inhibition percentage of 0.5 μM *Ath*DHAD-V496L as a function of aspterric acid (AA) concentration.

**Figure S17**. *Ath*DHAD-V496I mutant biochemical assay results. **A**. Reaction rate across different concentrations of α,β-dihydroxyisovalerate (DHI). **B**. Reaction rate across different concentrations of α,β-dihydroxymethylvalerate (DHMV). **C**. Inhibition percentage of 0.5 μM *Ath*DHAD-V496I as a function of aspterric acid (AA) concentration.

**Figure S18**. *Ath*DHAD-V496F mutant biochemical assay results. **A**. Reaction rate across different concentrations of α,β-dihydroxyisovalerate (DHI). **B**. Reaction rate across different concentrations of α,β-dihydroxymethylvalerate (DHMV). **C**. Inhibition percentage of 0.5 μM *Ath*DHAD-V496F as a function of aspterric acid (AA) concentration.

**Figure S19**. *Ath*DHAD-V497L mutant biochemical assay results. **A**. Reaction rate across different concentrations of α,β-dihydroxyisovalerate (DHI). **B**. Reaction rate across different concentrations of α,β-dihydroxymethylvalerate (DHMV). **C**. Inhibition percentage of 0.5 μM *Ath*DHAD-V497L as a function of aspterric acid (AA) concentration.

**Figure S20**. *Ath*DHAD-V497I mutant biochemical assay results. **A**. Reaction rate across different concentrations of α,β-dihydroxyisovalerate (DHI). **B**. Reaction rate across different concentrations of α,β-dihydroxymethylvalerate (DHMV). **C**. Inhibition percentage of 0.5 μM *Ath*DHAD-V497I as a function of aspterric acid (AA) concentration.

**Figure S21**. *Ath*DHAD-V497F mutant biochemical assay results. **A**. Reaction rate across different concentrations of α,β-dihydroxyisovalerate (DHI). **B**. Reaction rate across different concentrations of α,β-dihydroxymethylvalerate (DHMV). **C**. Inhibition percentage of 0.5 μM *Ath*DHAD-V497F as a function of aspterric acid (AA) concentration.

**Figure S22** *Ath*DHAD-I177L-V496L mutant biochemical assay results. **A**. Reaction rate across different concentrations of α,β-dihydroxyisovalerate (DHI). **B**. Reaction rate across different concentrations of α,β-dihydroxymethylvalerate (DHMV). **C**. Inhibition percentage of 0.5 μM *Ath*DHAD-I177L-V496L as a function of aspterric acid (AA) concentration.

**Figure S23**. *Ath*DHAD-I177L-V497L mutant biochemical assay results. **A**. Reaction rate across different concentrations of α,β-dihydroxyisovalerate (DHI). **B**. Reaction rate across different concentrations of α,β-dihydroxymethylvalerate (DHMV). **C**. Inhibition percentage of 0.5 μM *Ath*DHAD-I177L-V497L as a function of aspterric acid (AA) concentration.

**Figure S24**. *Ath*DHAD-V178L-V497L mutant biochemical assay results. **A**. Reaction rate across different concentrations of α,β-dihydroxyisovalerate (DHI). **B**. Reaction rate across different concentrations of α,β-dihydroxymethylvalerate (DHMV). **C**. Inhibition percentage of 0.5 μM *Ath*DHAD-V178L-V497L as a function of aspterric acid (AA) concentration.

**Figure S25**. 2-keto-isovalerate (KIV) calibration curve used for calculations of DHAD product concentration


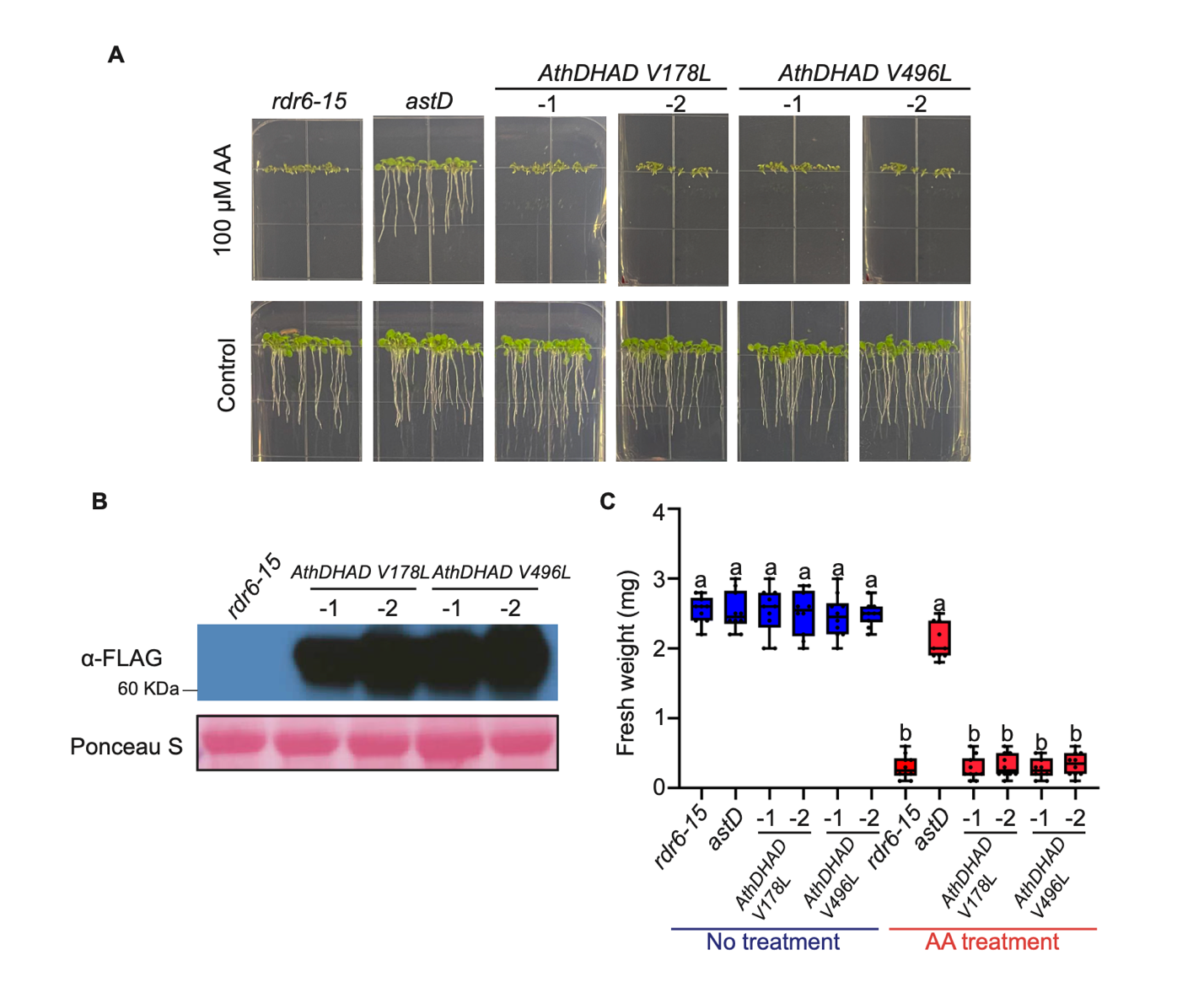


**Figure S26** Aspterric acid effect on *A. thaliana* carrying V178L or V496L mutant *Ath*DHAD.

**A**. Phenotype of seven-day-old *A. thaliana* on MS medium with (upper) and without (lower) 100 μM aspterric acid. Two independent transgenic lines for *pDHAD V178L* and *pDHAD V496L* were tested. The *astD* transgenic plants were used for positive control.

**B**. Immunoblot analysis of pDHAD V178L-2FLAG and pDHAD V496L-2FLAG protein levels in the indicated genotypes. Equal loading is shown by Ponceau S staining of a non-specifc band. Molecular mass marker in kilo Daltons is indicated on the left.

**C**. Fresh weight of seven-day-old *A. thaliana* seedlings growing on medium with (red) and without (blue) 100 μM aspterric acid. Error bars represent mean ± s.d (n=10).

3. REFERENCES

1. Goldschmidt, L.; Cooper, D. R.; Derewenda, Z. S.; Eisenberg, D., Toward rational protein crystallization: A Web server for the design of crystallizable protein variants. *Protein Sci* **2007,** *16* (8), 1569-76.

2. Yan, Y.; Liu, Q.; Zang, X.; Yuan, S.; Bat-Erdene, U.; Nguyen, C.; Gan, J.; Zhou, J.; Jacobsen, S. E.; Tang, Y., Resistance-gene-directed discovery of a natural-product herbicide with a new mode of action. *Nature* **2018,** *559* (7714), 415-418.

3.Kabsch, W., XDS. *Acta Crystallogr D Biol Crystallogr* **2010,** *66* (Pt 2), 125-32.

4. Otwinowski, Z.; Minor, W., Processing of X-ray diffraction data collected in oscillation mode. *Methods Enzymol* **1997,** *276*, 307-26.

5. McCoy, A. J.; Grosse-Kunstleve, R. W.; Adams, P. D.; Winn, M. D.; Storoni, L. C.; Read, R. J., Phaser crystallographic software. *J Appl Crystallogr* **2007,** *40* (Pt 4), 658-674.

6. Afonine, P. V.; Grosse-Kunstleve, R. W.; Echols, N.; Headd, J. J.; Moriarty, N. W.; Mustyakimov, M.; Terwilliger, T. C.; Urzhumtsev, A.; Zwart, P. H.; Adams, P. D., Towards automated crystallographic structure refinement with phenix.refine. *Acta Crystallogr D Biol Crystallogr* **2012,** *68* (Pt 4), 352-67.

7. Emsley, P.; Cowtan, K., Coot: model-building tools for molecular graphics. *Acta Crystallogr D Biol Crystallogr* **2004,** *60* (Pt 12 Pt 1), 2126-32.

8. Schrodinger, LLC, The PyMOL Molecular Graphics System, Version 1.8. 2015.

9. Humphrey, W.; Dalke, A.; Schulten, K., VMD: visual molecular dynamics. *J Mol Graph* **1996,** *14* (1), 33-8, 27-8.

10. Pettersen, E. F.; Goddard, T. D.; Huang, C. C.; Couch, G. S.; Greenblatt, D. M.; Meng, E. C.; Ferrin, T. E., UCSF Chimera--a visualization system for exploratory research and analysis. *J Comput Chem* **2004,** *25* (13), 1605-12.
